# Flow similarity, stochastic branching, and quarter-power scaling in plants

**DOI:** 10.1093/plphys/kiac358

**Published:** 2022-08-03

**Authors:** Charles A Price, Paul Drake, Erik J Veneklaas, Michael Renton

**Affiliations:** Department of Ecology and Evolutionary Biology, University of Tennessee, Knoxville, Tennessee 37996-3140, USA; School of Biological Sciences, University of Western Australia, Perth, Western Australia 6009, Australia; School of Biological Sciences, University of Western Australia, Perth, Western Australia 6009, Australia; School of Agriculture and Environment, University of Western Australia, Perth, Western Australia 6009, Australia; Centre of Excellence for Climate Change, Woodland and Forest Health, University of Western Australia, Perth, Western Australia 6009, Australia; School of Biological Sciences, University of Western Australia, Perth, Western Australia 6009, Australia; School of Agriculture and Environment, University of Western Australia, Perth, Western Australia 6009, Australia; Centre of Excellence for Climate Change, Woodland and Forest Health, University of Western Australia, Perth, Western Australia 6009, Australia; School of Biological Sciences, University of Western Australia, Perth, Western Australia 6009, Australia; School of Agriculture and Environment, University of Western Australia, Perth, Western Australia 6009, Australia; Centre of Excellence for Climate Change, Woodland and Forest Health, University of Western Australia, Perth, Western Australia 6009, Australia

## Abstract

The origin of allometric scaling patterns that are multiples of one-fourth has long fascinated biologists. While not universal, quarter-power scaling relationships are common and have been described in all major clades. Several models have been advanced to explain the origin of such patterns, but questions regarding the discordance between model predictions and empirical data have limited their widespread acceptance. Notable among these is a fractal branching model that predicts power-law scaling of both metabolism and physical dimensions. While a power law is a useful first approximation to some data sets, nonlinear data compilations suggest the possibility of alternative mechanisms. Here, we show that quarter-power scaling can be derived using only the preservation of volume flow rate and velocity as model constraints. Applying our model to land plants, we show that incorporating biomechanical principles and allowing different parts of plant branching networks to be optimized to serve different functions predicts nonlinearity in allometric relationships and helps explain why interspecific scaling exponents covary along a fractal continuum. We also demonstrate that while branching may be a stochastic process, due to the conservation of volume, data may still be consistent with the expectations for a fractal network when one examines sub-trees within a tree. Data from numerous sources at the level of plant shoots, stems, and petioles show strong agreement with our model predictions. This theoretical framework provides an easily testable alternative to current general models of plant metabolic allometry.

## Teaser

A general model for the origin of quarter-power scaling in land plants predicts allometry, allometric curvature, and allometric covariation within and across land plants.

## Introduction

Since Max Kleiber examined the scaling of animal metabolism with mass (Kleiber, 1932), scientists have been interested as to why allometric relationships often have exponents that are close to a multiple of one-fourth (Brown and West, 2000). Following the early works of Brody (1945) and Hemmingsen (1950), several seminal books published in the 1980s expanded the number and breadth of relationships that exhibit approximate quarter-power scaling, further generating interest in this area (McMahon and Bonner, 1983; Peters, 1983; Calder, 1984; Schmidt-Nielsen, 1984) and helping to establish a “mystery of quarter power scaling in biology” (Brown and West, 2000).

The publication of West, Brown, and Enquist’s (WBE) fractal branching model (West et al., 1997, 1999), which proposes a mechanism to explain the origin of quarter-power scaling relationships, further catalyzed interest in this area. WBE argued that a scaling relationship between organism volume, and the surface area available for resource exchange, should ultimately drive quarter-power scaling, and suggested that while external surface area in mammals could follow a geometric scaling (Rubner, 1883), internal vessel network geometry might be fractal, yielding quarter-power scaling and effectively giving life to a “fourth dimension” (West et al., 1999). Subsequent efforts to derive optimal network geometries invoke supply/demand arguments (Banavar et al., 1999, 2014), or volume minimization (Dodds, 2010). A common feature of these approaches is that they search for global optima and assume that fluid loss occurs at distributed sinks which are typically modeled as the ends of vessels. However, in both plants and animals, different parts of the fluid distribution network may be optimized to perform different functions (Murray, 1926; Price et al., 2013), and fluid is usually lost transmurally (Zwieniecki et al., 2002): vessel endpoints are not the usual mode of fluid exchange.

While the WBE model has been invaluable in helping to generate interest in biological scaling, unanswered questions regarding the disconnect between empirical data, model assumptions, and predictions have limited its widespread acceptance (Dodds et al., 2001; Niklas, 2004; Coomes, 2006; Price et al., 2007; Savage et al., 2008; Price et al., 2012). Several reports have indeed shown that proxies for metabolic rate in mature trees do scale with exponents close to the predicted three-fourth (Niklas and Enquist, 2001; Meinzer et al., 2005; Mori et al., 2010), but as recently highlighted by Price et al. (2012), empirical exponents by themselves do little to help determine an underlying mechanism. Given empirical support for a three-fourth scaling of metabolism, arguably the strongest subsequent test of WBE is whether or not the geometry of biological distribution networks conforms to the specific fractal structure that is invoked. Results from several studies suggest that while tree branching shows consistency with the assumed area-preserving architecture (see Eq. 1), the scaling of branch lengths is largely inconsistent with the WBE “space-filling” assumption (Price et al., 2011; Bentley et al., 2013; Tredennick et al., 2013).

Here, we suggest that plant distribution networks may indeed have “fractal-like” characteristics, but that these characteristics differ in important ways from those described by WBE. We show that a network which conserves volume flow rate and velocity, which we refer to collectively as “flow similarity,” also exhibits a three-fourth scaling relationship between surface area and volume. Subsequent incorporation of size-dependent hydraulic and biomechanical constraints leads to nonlinear predictions for numerous allometric patterns. To test this theory for the scaling of plant architecture and metabolism, we analyze the geometry of vascular plant networks at three levels of organization: (1) juvenile trees, (2) woody plant terminal stems, and (3) leaf petioles.

## Symmetric branching

We begin by considering the hydraulic behavior of the terminal branches in plants. Terminal branches in tracheophytes generally, and in woody species in particular, are where the overwhelming majority of leaves are borne and thus constitute the predominant sites of photosynthesis and production. We follow previous work (Shinozaki et al., 1964; West et al., 1997, 1999; Savage et al., 2010) in modeling branching as an idealized, symmetric, branching flow network. For now, we assume that locally, the number of internal conduits scales linearly with the number of external branches, $n_{\mathrm{int}}\propto n_{\mathrm{ext}}^{p}$, where *p* ≈ 1. Under such an assumption, the scaling of the internal vessels parallels that of the external branches thus, for the purposes of the following derivation, we do not differentiate between the two. We consider exceptions to this assumption in the “Discussion” and Supplemental Note 1.

Under symmetric branching, the ratio of the daughter (*k + 1*) to parent (*k*) branch radii is *r_k+1_/r_k_ = n^−a^*, where *n* is the number of daughter branches. If we assume that flow velocity is constant across branching generations, we have area preserving branching where *a* = 1/2, and thus

(1) $$r_{k+1}={n^{-1/2}r}_{k}$$

Volumetric flow rate (*Q*) through a conduit within the network can be approximated via the well-known Hagen–Poiseuille equation as $Q=\frac{\pi r^{4}\left|\Delta P\right|}{8\eta l}$, where *r* is conduit radius, *l* is conduit length, Δ*P* is the difference in pressure between the ends of the conduit, and *η* is viscosity. If we assume that *η* and Δ*P* are locally constant (the same in parent and daughter branches, see Supplemental Note 1), then we have

(2) $$l_{k}=\frac{C{r_{k}}^{4}}{Q_{k}}$$

and

(3) $$l_{k+1}=\frac{{{Cr}_{k+1}}^{4}}{Q_{k+1}}$$

where *c* = $\frac{\pi \left|\Delta P\right|}{8\eta }$ and thus (from Eq. 2) the ratio of radius squared to length in the parent branch is

(4) $$\frac{{r_{k}}^{2}}{l_{k}}={r_{k}}^{2}\frac{Q_{k}}{C{r_{k}}^{4}}=\frac{Q_{k}}{{{Cr}_{k}}^{2}}$$

Furthermore, with symmetry the volumetric flow from the parent branch is divided evenly among its daughters, and so

(5) Qk+1=Qkn=n-1Qk

Based on Equation 4, the ratio of radius squared to length in the daughter branch is

(6) $$\begin{array}{l} \frac{r_{k+1}{}^{2}}{l_{k+1}}=\frac{Q_{k+1}r_{k+1}{}^{2}}{Cr_{k+1}{}^{4}}=\frac{Q_{k+1}}{Cr_{k+1}{}^{2}}\\ =\frac{n^{-1}Q_{k}}{C{\left(n^{-1/2}r_{k}\right)}^{2}}\;(\text{based}\;\text{on}\;\text{Eqs}\;1\;\text{and}\;5)\\ =\frac{n^{-1}Q_{k}}{Cn^{-1}r_{k}{}^{2}}=\frac{Q_{k}}{Cr_{k}{}^{2}}\\ =\frac{r_{k}{}^{2}}{l_{k}} \end{array}$$

This means that the ratio of radius squared to length is the same in the daughter and parent branch, and since this can be shown for any daughter/parent branch combination within the local structure, we have the general relationship

(7) $$l\propto r^{2}.$$

Using standard formulas for the surface area, SA=2πrl and volume,$\;V=\pi r^{2}l$, of a cylinder, together with Equation 7, we have $SA\propto 2\pi r^{3}$ and$\;V\propto \pi r^{4}$, and so

(8) $$SA\propto V^{3/4}.$$

These scaling arguments apply to the individual internodes within a tree, but in Supplemental Note 2 we show that the *l ∝ r*^2^ and *SA ∝ V*^3/4^ scaling result can be extended to sub-trees, and indeed the entire tree. Additional relationships between the length, diameter, surface area, and volume in fractal trees follow easily from Eqs. 7 and 8 (Table 1). Thus, only two physical principles, the conservation of volumetric flow rate and velocity across the hydraulic network are required to derive a three-fourth relationship between surface area and volume at the level of both individual internodes and whole tree structures. If bulk tissue density is constant across branches locally, and metabolic rate is proportional to leaf area, which is in turn proportional to stem surface area, a three-fourth relationship between metabolism and mass emerges.

**Table 1:** Predicted and observed relationships between length, diameter, surface area, and volume

Y-variable	Length	Surface Area	Diameter	Length	Diameter	Length
X-variable	Diameter	Volume	Volume	Volume	Surface area	Surface area
Expression	L = D^α^	SA = V^(α+1)/(α+2)^	D = V^1/(α+2)^	L = V^α/(α+2)^	D = SA^1/(α+1)^	L = SA^α/(α+1)^
Flow Similarity	2	3/4	1/4	1/2	1/3	2/3
Elastic Similarity	2/3	5/8	3/8	1/4	3/5	2/5
Changing Exponent	2 to 2/3	3/4 to 5/8	1/4 to 3/8	1/2 to 1/4	1/3 to 3/5	2/3 to 2/5
Curvature	Concave	Concave	Convex	Concave	Convex	Concave
Tree Data Slope (raw data) (588)	−1.639	0.749	0.389	0.638	0.524	0.858
Tree Data Slope Confidence Intervals	−1.777 to −1.511	0.723 to 0.763	0.370 to 0.410	0.599 to 0.679	0.489 to 0.562	0.823 to 0.896
Tree Data *R*^2^	0	0.888	0.593	0.395	0.263	0.726
Tree Data Slope (sub-trees) (588)	2.06	0.798	0.314	0.648	0.394	0.813
Tree Data Slope CI's	1.998 to 2.131	0.791 to 0.805	0.308 to 0.321	0.635 to 0.662	0.384 to 0.404	0.802 to 0.823
Tree Data *R*^2^	0.842	0.989	0.928	0.932	0.901	0.973
Stem Data Slopes (436)	1.978	0.763	0.288	0.567	0.376	0.743
Stem Data Slope CI's	1.84 to 2.126	0.751 to 0.776	0.278 to 0.298	0.542 to 0.593	0.358 to 0.396	0.720 to 0.766
Stem Data *R*^2^	0.506	0.972	0.879	0.769	0.768	0.892
Petiole Data Slopes (955)	1.979	0.759	0.282	0.558	0.372	0.735
Petiole Data Slope Confidence Intervals	1.88 to 2.084	0.751 to 0.767	0.274 to 0.29	0.542 to 0.574	0.358 to 0.386	0.721 to 0.749
Petiole Data *R*^2^	0.348	0.972	0.797	0.793	0.648	0.91

*Y* and *X* variables are listed in the top two rows. An expression for each relationship is in the third row, where *α* is the length to diameter exponent which is equal to 2 under flow similarity. Rows 4–7 represent the predictions for flow similarity, elastic similarity, the change in exponent expected going from small to large plants (i.e. from flow to elastic similarity), and the expected curvature from such a relationship. Rows 8–22 represent the observed slopes, 95% slope confidence intervals and *R*^2^ for each relationship, for the tree data, tree data sub-trees, stem data, and petiole data.

## Self-loading

Next, we consider the addition of biomechanical theory to meet the demands of self-loading. Theory linking tree height to stem diameter has long been established based on Euler’s buckling model (McMahon and Kronauer, 1976; Niklas, 1994), which predicts that the maximum height (*l*_max_) to which an idealized column can be extended scales with its radius (*r*) as

(5) $$l_{\max}=c{\left(\frac{E}{\rho g}\right)}^{1/3}2r^{2/3},$$

where *E* is the modulus of elasticity, *ρ* is bulk tissue density, *g* is the acceleration due to gravity, and *c* is a proportionality constant. *E*, *g*, and *ρ* are frequently assumed to be constant (Niklas, 1994) leading to *l*_max_*∝ r*^2/3^. Many studies have evaluated elastic similarity in large trees and found empirical support, particularly in the larger branches (Holbrook and Putz, 1989; West et al., 1999). However, nonlinearity is also a common feature of empirical data. For example, plots of plant height versus stem diameter are frequently concave on logarithmic axes with slopes typically steeper than the predicted two-thirds at small size scales (Bertram, 1989; Niklas, 1995; Niklas and Spatz, 2004; Muller-Landau et al., 2006; Enquist et al., 2007) and one theoretical effort (which assumes a three-fourth scaling of growth with metabolism) predicts that hydraulics, not biomechanics, determines the two-thirds scaling of height with diameter (Niklas and Spatz, 2004).

We propose that different parts of the tree branching system may be subject to different physical constraints, with small plants, or the peripheral branches of large trees where biomechanical demands are minimal, more consistent with flow similarity (*l ∝ r*^2^) , and the basal branches of large trees more consistent with elastic similarity (*l ∝ r*^2/3^). In a symmetric bifurcating tree, the increase in branch numbers is proportional to 2^*n*^ where *n* is the number of branching generations. Peripheral branches thus are expected to exhibit a strong influence on the allometric slopes of some relationships due to their relative abundance. The exact contribution of each branching generation to the overall scaling exponent depends on tree size and the nature of the *l ∝ r*^2^ to *l ∝ r*^2/3^ transition (linear or nonlinear), the number of branching generations, and the degree of side branching. These factors will differ between species and will ultimately require detailed simulations (Smith et al., 2014; Brummer et al., 2017) and/or empirical measurements to determine. Allowing the length–radius scaling to vary within a tree, between trees of differing size, or between species predicts nonlinearity in allometric relationships. Table 1 lists predicted curvatures (convex or concave) for each of the six relationships examined here. Representing the length versus radius scaling as *l ∝ r^α^*, one can predict a continuum of variability for each allometric relationship (Table 1), and covariation functions for each pairwise combination of exponents (Supplemental Table S1), all of which are the function of a single parameter (*α*). Linear regression fits to curved data will often have slopes that fall between the flow similarity and elastic similarity expectations; however, the expected value of the slope will depend strongly on the size range, side branching, whether or not there is systematic variation in species wood density, and how evenly each size class is sampled.

## Asymmetric branching

The above theory predicts the dimensions of idealized symmetric branching networks. Real networks, however, are usually not so orderly, and branching is commonly asymmetric (Smith et al., 2014; Brummer et al., 2017). Insight can be gained by considering the probability of a branching event. For fractal networks, that probability is scale-free and results in a power-law distribution of both lengths and radii. In contrast, biological networks typically involve the acquisition or distribution of resources that occur over finite, and probably characteristic length scales. For example, it has recently been shown that leaf vein networks have frequency distributions of vein radii that are well approximated by a power law, and distributions of vein lengths that are better fit by an exponential distribution, suggestive of a characteristic scale (Price et al., 2011). Hence, one can model branching events as a stochastic process with constant branching (or stopping) probability *p*, leading to $P(l)=pe^{-pl_{c}}$, with average length or characteristic scale *l_c_* equal to 1*/p.*

Modeling branch lengths as a stochastic process raises the question of how empirical data might follow the predictions developed for a fractal network if they also branch stochastically, that is, asymmetrically. In contrast to previous approaches, the model herein does not require volume to be conserved globally across all branching generations (i.e. $\sum \pi l_{k}r_{k}^{2}$ need not equal $\sum \pi l_{k+1}r_{k+1}^{2}$). Rather we derive predicted relationships between the basal radius of a sub-tree and its length, surface area, and volume, which should hold for all sub-trees within a branching structure regardless of symmetry. Therefore, by examining sub-trees within a network (Bentley et al., 2013), that is, treating each as an individual tree, one can test whether whole trees conform to the expectations developed for ideal symmetric networks as we illustrate below.

## Results

**Figure 1:**
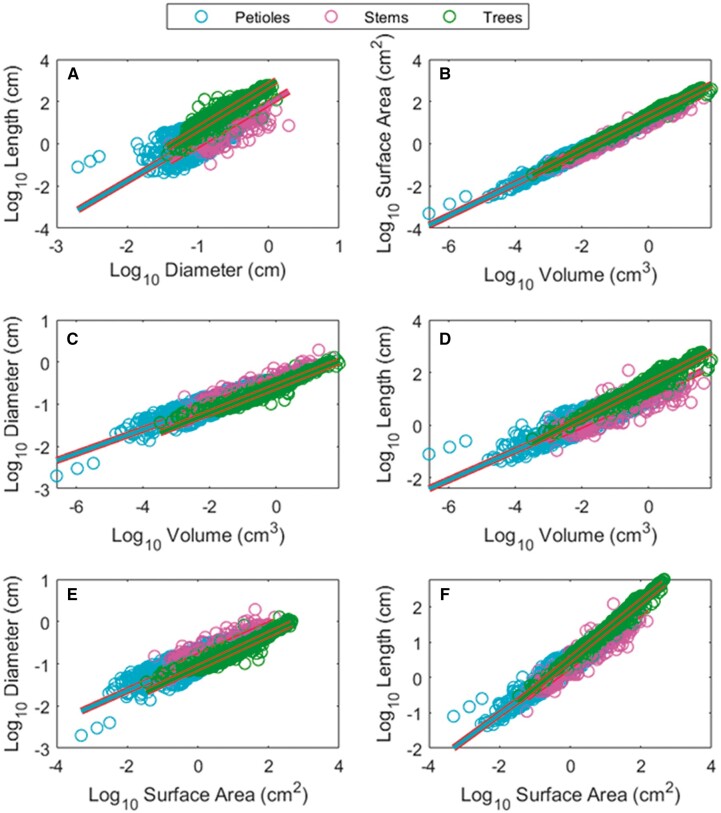
Allometric relationships among plant branch dimensions. Bivariate plots of the dimensions of the tree data, stem data, and petiole data, plotted on a common axis for pairwise relationships between length, diameter, surface area, and volume (A–F). SMA regression statistics for all relationships are presented in Table 1. Tree data are based on sub-trees as described in the “Materials and methods”. While length–diameter relationships are characterized by lower coefficients of determination (*R*^2^), surface area–volume relationships are tightly correlated.

Standardized major axis (SMA) regression results for the data presented in Figure 1 are in Table 1. Slopes are closer to flow similarity than elastic similarity (WBE) predictions in all cases. The confidence intervals for many of the relationships are quite narrow due to the high *R*^2^ values and do not always include the predictions of the flow similarity model, but none include the elastic similarity predictions. Note the substantial increase in *R*^2^ values when comparing slopes fit to the raw tree data and those fit to sub-tree data (rows 8 and 11, respectively, in Table 1).

**Figure 2:**
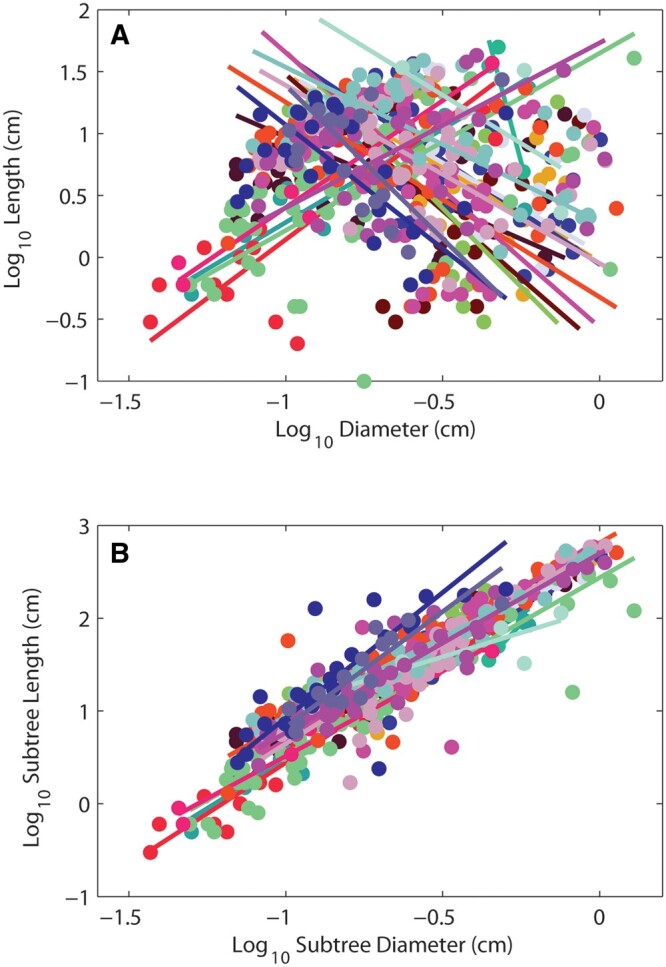
Length versus diameter in raw data and sub-trees. Allometric relationships between stem internode length and diameter in raw tree data (A), and the same relationship in sub-trees within each of 19 trees (B, see “Materials and methods”). The raw data correlations are highly variable with both positive and negative slopes (mean slope = −0.95 and slope standard deviation 1.54); however, sub-tree regressions converge toward the predicted value of 2 (mean sub-tree slope = 2.05 and slope standard deviation = 0.24). This demonstrates how by examining sub-trees within a tree can help determine if branching conforms to the expectations developed for symmetric fractal trees.

Figure 2A demonstrates how individual branch internode lengths (tree data) and their corresponding basal diameters are poorly correlated (mean *R*^2^ of 0.198) with slopes that are both positive and negative. However, if instead of internode length, one examines the total length of all branches distal to a given branch internode (Figure 2B), and the diameter of that branch internode, the slopes for the relationships tighten considerably around a mean value of 2.05 (mean *R*^2^ of 0.875). Supplemental Figures S1–S19 show all six pairwise relationships for each individual tree, both as raw data and as sub-trees. The mean *R*^2^ for the raw data across all pairwise regressions was 0.48 while the mean *R*^2^ for the sub-trees was 0.94 (Supplemental Table S2). Additional statistics for individual and species level regressions for the tree data are presented in Supplemental Tables S2 and S3, respectively.

**Table 2:** Exponential versus power-law comparison

Organ	Species	Dimension	Exponential	Power law	AICc_P	AICc_E	Relative likelihood	Sample size
Tree branches	*Eucalyptus gomphocephela*	Lengths	Y		1.61E + 03	1.33E + 03	1.33E − 61	203
Tree branches	*Eucalyptusgomphocephela*	Diameters		Y	−91.78	−88.09	0.16	203
Tree branches	*Eucalyptus caesia*	Lengths	Y		629.75	541.61	7.25E − 20	94
Tree branches	*Eucalyptus. caesia*	Diameters		Y	−105.09	−97.55	0.02	94
Tree branches	*Eucalyptusdiversicolor*	Lengths	Y		1.66E + 03	1.38E + 03	2.13E − 60	203
Tree branches	*Eucalyptus diversicolor*	Diameters		Y	−111.22	−108.38	0.24	203
Tree branches	*Eucalyptus incrassata*	Lengths	Y		663.87	581.04	1.03E − 18	88
Tree branches	*Eucalyptus incrassata*	Diameters		Y	−123.24	−78.83	2.27E − 10	88

For the data set that contained full hierarchical trees (tree data), each species was tested to determine if the distribution branch segment lengths and diameters were better fit by an exponential model (column 4) or a power-law model (column 5). In all cases, length distributions were better fit by an exponential model, and diameter distributions better fit by a power law (indicated by the letter “Y”). Columns 6 and 7 represent the size corrected Akaike’s information criterion score for the power law and exponential models respectively, with their relative likelihood in column 8 followed by the sample size.

**Figure 3:**
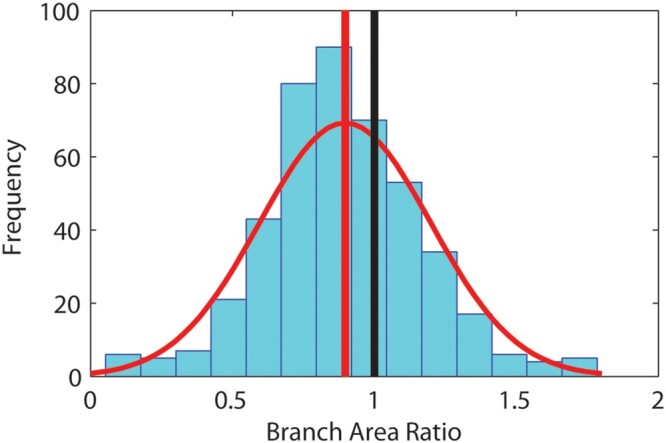
Frequency distributions for tree data branch area ratios. The distribution is well approximated by a normal curve and includes the expectation for area-preserving branching (black vertical line). The mean tree data branch area ratio (red vertical line) is slightly lower than the expected value.

**Figure 4:**
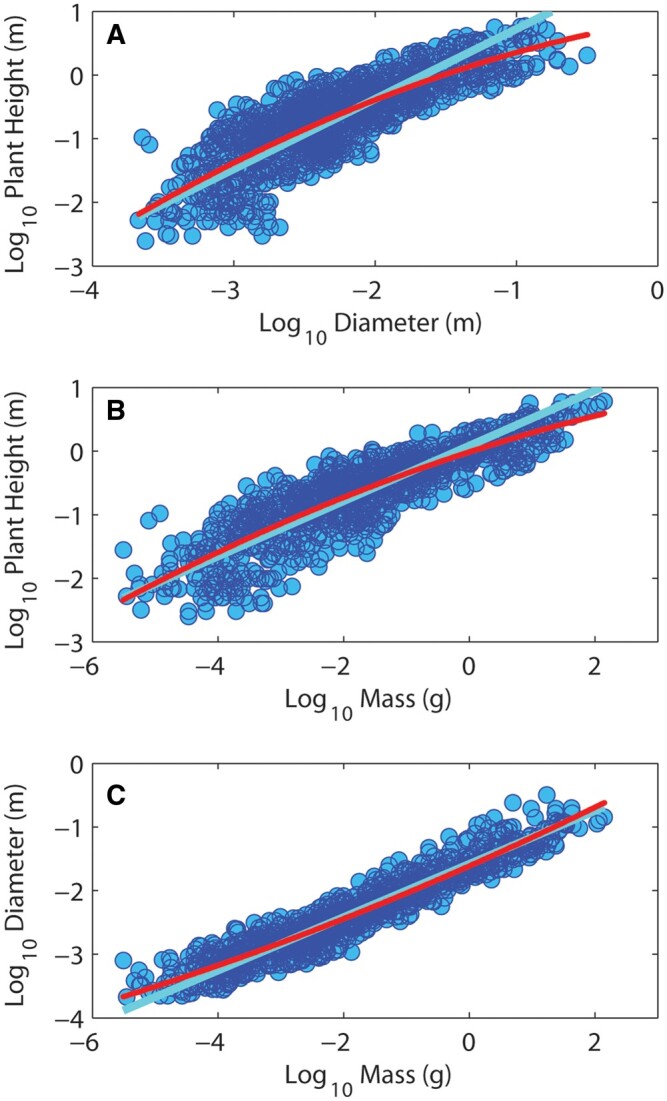
Allometric relationships between plant height, basal stem diameter and above ground plant mass from a compilation of Sonoran Desert plant allometric data. Plant height versus basal diameter (A), height versus mass (B), and basal diameter versus mass (C). The red line in each plot represents a second-order polynomial fit to the data to determine the curvature, and the light blue line represents SMA regression fit. Polynomial curvatures are consistent with those predicted (Table 1), concave in subparts (A and B), and convex in C. Regression slopes for A (1.1) and B (0.43) fall in between flow and elastic similarity predictions as might be expected, and the slope for C (0.42) is just outside of this range (see Table 1).

Table 2 shows that for the tree data, frequency distributions of lengths and diameters are always better fit by exponential and power-law models, respectively. Figure 3 shows the frequency distributions of area ratios for the tree data, which is well approximated by a normal curve and strongly overlaps the expectation for area preserving branching. Figure 4 shows that for allometric relationships between plant height, basal stem diameter, and above-ground plant mass, polynomial fits to data display curvature consistent with that predicted in Table 1. SMA regression statistics for the three relationships are in Supplemental Table S4.

## Discussion

Collectively, the data presented herein demonstrate that: (1) scaling relationships consistent with $l\propto r^{2}$ and $SA\propto V^{3/4}$ are found throughout aboveground plant branching networks; (2) area preserving branching is common, consistent with earlier reports (Horn, 1971; Bentley et al., 2013); (3) the frequency distributions of branch lengths are consistent with the expectations of a Poisson process; and (4) by examining sub-trees within a tree, one can determine if branching is consistent with expectations developed for theoretical symmetric fractal networks. Taken together, the theory and data presented provide support for a three-fourth scaling of surface area to volume in plant branching architecture as a potential driver of metabolic scaling patterns across plants, and we propose a mechanism, flow similarity, that differs from earlier modeling efforts (West et al., 1999).

If basal parts of the branching pathway in plants serve a greater biomechanical role, departure from exact flow similarity model predictions is expected given that allometric relationships will exhibit some nonlinearity. While it is possible to predict the direction of the curvature (convex or concave), predicting the exact function, or the slopes of linear models fit to curvilinear data, is challenging, as it will depend on plant size, branch size and attendant biomechanical demands, the degree of side branching, and whether or not there is systematic variation in bulk density. This may explain why observed slopes are slightly higher than predicted values for some of the relationships in Table 1 (columns 4–7). For example, the slopes of lines fit to data in the form of convex curves that have positive first derivatives everywhere, will usually fall between the minimum and maximum derivative values, that is between flow similarity and elastic similarity predictions.

The theoretical approach described herein has several advantages over its predecessors. It is a single parameter model that is consistent with known mechanisms of leaf display (along branches, not just at branch tips), and of fluid loss from xylem (transmural flow) (Zwieniecki et al., 2002). The model also operates both at the level of branch internodes and across sub-trees within a tree, thus it is relatively easy to evaluate empirically (Supplemental Note 2). Incorporating both flow similarity and elastic similarity into a common framework helps to explain the curvature common to many allometric data sets (Bertram, 1989; Niklas, 1995; Niklas and Spatz, 2004; Muller-Landau et al., 2006; Enquist et al., 2007), why linear fits to curvilinear data fall between the different model predictions, and why interspecific allometric relationships covary (Price et al., 2007). However, the specific covariation functions described here (Supplemental Table S1) differ from those predicted in earlier work (Price et al., 2007). This is because the predicted branch dimensions differ between the models, and because under flow similarity, surface area is proportional to branch surface area, whereas under the Price et al. (2007) approach (based on the WBE framework), surface area is proportional to the number of terminal branches.

The model developed here is an idealized abstraction, much like its conceptual predecessors (Shinozaki et al., 1964; West et al., 1997, 1999; Niklas and Spatz, 2004; Savage et al., 2010); however, our model differs in that we view our model as a species level model, but one that can be applied to any species that more or less conforms to its simplifying assumptions. Tests of the model will generally be more valid at the species or genus level, rather than at the family level or higher. For example, tests examining allometric relationships within a species (i.e. terminal stems or sub-trees) or interspecific collections of intraspecific allometric relationships (as in our tree data, Supplemental Tables S2 and S3) will likely be more informative because the simplifying assumptions will be closer to being valid. Empirical validation of the model will depend strongly on the trait in question (i.e. bulk density, leaf size, or stem-specific conductivity), the amount of trait variance for the clade in question, and whether such variance changes systematically with plant size (Price et al., 2014).

The theory assumes that leaf area is proportional to stem surface area in terminal branches, but in many species, leaves are ephemeral and thus total leaf area produced over a growing season, or leaf scars, might be more tightly correlated with stem surface area rather than the leaf area or number of leaves found at any one time.

Published data for the logarithmic relationship between above ground dark respiration and plant mass suggest that the scaling is isometric at small sizes (Reich et al., 2006) shifting to a slope close to three-fourth at large sizes (Mori et al., 2010). This may result from the fact that in small seedlings and saplings, most or all tissue is metabolically active, and total respiration is not limited by the stem surface area available for leaf display. However, as trees grow larger, an increasing proportion of a trees’ total mass is composed of tissue that has low or no metabolic activity, and respiration will increasingly be dominated by leaves, and to a lesser extent, the sapwood, the active cambium layer and the phloem, all of which are expected to be approximately proportional to branch surface area, causing empirical slopes to shift towards the theoretical relationships described herein.

Velocity preservation and the conservation of volume flow rate are intuitive and arguably parsimonious model constraints. However, more integrative traits that reflect network efficiency may be the targets upon which natural selection is ultimately acting. For example, for a given *Q* and Δ*P*, and a constant sapwood area fraction, a branching that follows $l\propto r^{2}$ would conserve sapwood specific conductivity (*K_S_*), defined as $K_{S}=\frac{Ql}{\Delta Pr_{s}^{2}},\;$where *r_s_* is the sapwood radius. While space precludes a detailed exploration of variability in stem conductivity within and across tree branches, there is evidence to suggest that in the absence of environmentally driven variation, or branch order/path length-dependent effects, sapwood-specific conductivity may be a conserved species-specific trait (McDowell et al., 2002; Sellin et al., 2008). Specific conductivity need not be conserved throughout the entire plant branching network for flow similarity to have a strong influence on the allometry of metabolism, again due to the numerical dominance of the terminal parts of the branching pathway.

Area preserving branching has strong empirical support among external tree branches (Horn, 2000; Bentley et al., 2013), and across vessel bundles in leaves (Price et al., 2013), but the physical processes underlying this principle are not yet fully established. Published data suggest that velocity can increase, decrease, or not vary statistically as a function of branch diameter within and across species (McCulloh and Sperry, 2005; Savage et al., 2010). Thus, the extent to which velocity preservation can be invoked as a global constraint remains an open question. It is easy to envision that sap velocity may be more constrained locally as rapid changes in velocity over short distances would seem disadvantageous. Indeed, velocity measures for similar sized branches within the same species are often quite close to one another (Savage et al., 2010). In a recent summary of existing data, Savage et al. (2010) found no significant relationship between branch diameter and maximum sap velocity in 8 out of 12 species. Considering the relatively low number of species for which these variables have been measured, the relationships between branch geometry and fluid dynamics warrant further inquiry.

We have invoked a linear relationship between internal and external branching locally as a simplifying model assumption. However, tapering of conduit dimensions is well supported empirically at the scale of entire trees (Ewers and Zimmerman, 1984; Anfodillo et al., 2006; Coomes et al., 2007; Sellin et al., 2008; Savage et al., 2010), and this would imply a nonlinear relationship between internal and external branching (Savage et al., 2010). It can be shown that our model, with its assumptions of area-preserving branching and flow similarity, and its data-supported prediction of volume to surface-area scaling are consistent with conduit dimension tapering if the pressure drop across branching generations varies in a certain way from parent to daughter branches (Supplemental Note 1).

A key difference between our approach and earlier attempts based on “space-filling” fractal networks is with respect to the scaling of branch lengths. As discussed in Price et al. (2012), “space-filling” evokes a branching structure that fills a 3D geometric space; however, as defined by WBE (1997, 1999), “space-filling” simply means that the sum of the volumes supplied by a conduit, that have a volume radius that is equal to the length of that conduit, are constant for conduits of a particular order ($N_{k}l_{k}^{3}=N_{k+1}l_{k+1}^{3}$). For symmetric bifurcating networks that also have area-preserving branching, this results in a specific ratio between parent and daughter branches *l_k+1_*/*l_k_* ≈0.794, and a scaling of length with radius that is assumed to follow elastic similarity ($l\propto r^{2/3}$). Such a network is “scale-free” meaning that frequency distributions of conduit lengths and radii should exhibit power-law behavior. Subsequent modifications to allow for side-branching have generally retained this “space-filling” assumption (Smith et al., 2014; Brummer et al., 2017); however, some WBE “extensions” that demonstrate allometric covariation among empirical scaling exponents have allowed length and radius ratios to vary (Price and Enquist, 2006, 2007; Price et al., 2007).

Our approach differs in several important ways from these earlier attempts. Tree branches display leaves to intercept light, and branching events are known to be catalyzed by light availability (Leduc et al., 2014; Schneider et al., 2019). Light will be heterogeneously distributed within a canopy due to self-shading and shading from other species. We infer that for a given species there is an average length scale associated with the 3D distribution of light levels required to cause a branching event and that branching probabilities will thus begin to look like a Poisson process as the number of branching events in trees grows. Because volume need not be conserved across branching generations in our model, abundant side branching to acquire heterogeneously distributed light is expected, yet trees can still conform to the self-similar expectations we have developed because the relationships only need be locally conserved (i.e. sub-trees, Supplementary Note 2). Thus, in contrast to “space-filling” approaches that are in fact scale-free, we expect branching to have a characteristic length scale associated with the gathering of spatially distributed resources.

Another key difference is that earlier fractal branching models (West et al., 1997, 1999) invoke resource exchange (with leaves) only at network ends and that the three-fourth exponent emerges only as the network grows to an infinite size (Savage et al., 2008) requiring corrections for finite sized networks that yield an exponent closer to 0.81. Our model allows for leaf display along branches and the three-fourth scaling of surface area to volume can emerge at the level of a single bifurcation, or ontogenetically for a single growing branch. The approximate three-fourth scaling of branch surface area to volume appears to have strong empirical support in our data. Subsequent characterization of this pattern in other clades and larger trees is required to understand the full scope of this potential mechanism. We offer the maintenance of flow similarity as one possible mechanism, which may drive a three-fourth scaling of metabolic rate with mass, particularly if the terminal branches in plants have a strong influence on metabolic scaling exponents but recognize that empirical support is needed for the simplifying assumptions of velocity preservation and a constant pressure drop, or variable pressure drop with conduit tapering (Supplemental Note 1). Subsequent sensitivity analyses exploring the relaxation of these assumptions will help to determine how they potentially influence plant geometry and metabolic scaling patterns.

If flow similarity, as reflected in *l ∝ r*^2^ scaling, underlies or contributes to quarter-power scaling in plants, it is natural to speculate as to why it has remained hidden. This may be due to several factors. First, because of the stochastic nature of branching, raw plots of branch length versus diameter measures will exhibit poor correlations with highly variable slopes, effectively obscuring the *l ∝ r*^2^ signal that may be revealed by examining sub-trees (Figure 2; Supplemental Figures S1–S19). Second, a focus on the scaling of tree height with stem diameter in large trees (McMahon and Kronauer, 1976; West et al., 1999) may have driven a search for explanations that exhibit only *l ∝ r*^2/3^ scaling, which may apply to large branches or tree height in large trees, but does not explain the *l ∝ r*^2^ scaling in abundant branch ends, or the nonlinearity common to many data sets: total plant height and total branch path length are both qualitatively and quantitatively different phenomena (Niklas and Spatz, 2004). Lastly, most theoretical attempts have searched for global optima, not allowing for the fact that different parts of networks may be optimized to perform different functions (Price et al., 2013).

Some have questioned whether allometric patterns in biology such as “Kleiber’s law” are even laws at all, noting the variability in both intraspecific and interspecific scaling exponents observed in empirical data (Dodds et al., 2001; Glazier, 2006). Large collections of interspecific data, and several meta-analyses of intraspecific data have shown that empirical data often (but not always) have slopes that cluster around values close to the canonical three-fourth (Niklas and Enquist, 2001; Brown et al., 2004; Savage et al., 2004; Mori et al., 2010) albeit with curvature in some data sets (Savage et al., 2004; Mori et al., 2010). Questions of whether or not collections of allometric data showing scaling exponents near one-fourth constitute a “biological law” are largely semantic in nature and not easily answered to the satisfaction of all. Perhaps a more productive approach would be to ask whether scaling exponents that are some multiple of one-fourth are common enough that they might emerge from a shared mechanism such as that described herein.

## Materials and methods

The model described herein makes predictions for geometric, hydraulic, and biomechanical scaling relationships in plants and thus numerous tests to evaluate its predictions could be envisioned. We focus here on the network geometry as a first test for the simple reason that if the geometric predictions are not met, subsequent tests of hydraulic or biomechanical predictions are less relevant. To do so we evaluate the allometry of network dimensions in the branches of whole tree saplings, terminal stems, and petioles (described below). All individual plant stems, or petioles were approximated as cylinders based on their length and diameter, with surface area and volume for each approximated using standard geometric formulas. The predictions from the above theory were then evaluated in four ways: (1) by examining SMA regression slopes fit to bivariate relationships between length, diameter, surface area, and volume and comparing slopes to flow and elastic similarity model predictions; (2) by examining the frequency distributions of branch lengths and diameters to determine if they are better fit by an exponential or power-law model (applicable to “plant data” described below); (3) by examining the daughter/parent branch area ratios (applicable to “plant data”); and (4) examining the curvature in length–mass–diameter relationships in a large plant allometric data set. To compare like with like, surface area under elastic similarity was evaluated as the surface area of a cylinder following elastic similarity ($l\propto r^{2/3}$), not proportional to the number of terminal branches as in WBE.

The four data sets used to evaluate the model are described below. In the interest of clarity we will refer to these as “tree data”, “stem data”, “petiole data”, and “allometric data” throughout.

### Tree data

The length, diameter, and connectivity of all stems greater than 1 mm were measured in 19 individual saplings, from four species all within the *Eucalyptus* genus, in the family *Myrtaceae*. The species, with number of individuals in parentheses, were *Eucalyptus gomphocephala* (6), *Eucalyptus caesia* (5), *Eucalyptus diversicolor* (4), and *Eucalyptus incrassata* (4). These individuals were grown from seed for 2 years under light shade on the University of Western Australia campus prior to harvest. Individuals were selected to span as wide a range of intraspecific size as possible, with the number of branches per individual ranging from 3 to 59 with a mean of ∼31. These data were then examined as bivariate relationships between the individual stem internode dimensions at the individual level, at the species level, and across all species. Data were analyzed as “raw data”, that is, individual branch internodes, and also within all possible “subtrees”, where a sub-tree is defined as the diameter of a given branch internode, and the total length, total surface area, and total volume of all branch segments distal to that branch internode.

### Stem data

Terminal stems, defined for this data set as all stem internodes distal to the bud scar from the previous year, were collected from 122 species from the *Banksia* genus (referred to as “stems” throughout) in August 2012. Most species were represented by a single stem, but several species had multiple stems, with a maximum of 44 stems (*B. hewardiana*). Stems with minimal damage or evidence of herbivory and growing in full sunlight were selected. All *Banksia* stems were collected from the Banksia Farm (www.banksiafarm.com.au), a private arboretum containing almost all known members of the *Banksia* genus and are thus effectively from a common garden. The Banksia Farm is located in Mount Barker (34°37′48″S, 117°40′1″E), situated ∼370 km south of Perth, Western Australia. Mount Barker is characterized by a temperate climate, with an annual average high temperature of 20.1°C, an average low of 9.4°C, and a mean annual precipitation of 725 mm.

### Petiole data

A total of 935 individual leaves from 43 temperate angiosperm species were collected as part of a leaf allometric study in 2007–2008 (full description in Price et al., 2009) for which the petiole dimension data was not analyzed or published. For each species, between 18 and 40 (mean = 21.7) individual leaves of increasing size were collected and the length and diameter of their petioles recorded.

### Allometric data

The Sonoran Desert allometric data set comprised plant height, basal stem diameter, and aboveground dry mass for 1,504 individuals from 63 species all found growing in the Sonoran Desert region of the southwestern USA. These data were previously analyzed to evaluate covariation in intraspecific allometric relationships, but the interspecific relationships described herein were not published (for a full description, see Price et al., 2007).

### Statistics

All bivariate relationships were log-transformed prior to analyses. We used SMA regression in the software package SMATR to estimate the slopes for all relationships as is common practice in allometric analyses (Warton et al., 2006). Frequency distributions of internode lengths and diameters for the tree data were fit with exponential and power-law models following Price et al. (2011). To compare the exponential and power-law model fits, we used the method of maximum likelihood to estimate the model parameters and likelihood. We then used a sample size corrected Akaike’s information criterion to compare models, AICc = AIC + (2*k*(*k* + 1))/(*n* − *k* − 1) (Burnham and Anderson, 2002), where *k* is the number of model parameters, which is 1 for the exponential model, and 2 for the power-law model, and *L* is the likelihood. Table 2 reports corrected AIC scores for the exponential (AICc_E) and power-law models (AICc_P) and their relative likelihood, which is the probability that the model with the lower AICc score minimizes information loss (Burnham and Anderson, 2002).

## Supplemental data

The following materials are available in the online version of this article:

**Supplemental Figure S1.** Allometric relationships for *E. gomphocephela* sample 1.

**Supplemental Figure S2.** Allometric relationships for *E. gomphocephela* sample 2.

**Supplemental Figure S3.** Allometric relationships for *E. gomphocephela* sample 3.

**Supplemental Figure S4.** Allometric relationships for *E. gomphocephela* sample 4.

**Supplemental Figure S5.** Allometric relationships for *E. gomphocephela* sample 5.

**Supplemental Figure S6.** Allometric relationships for *E. gomphocephela* sample 6.

**Supplemental Figure S7.** Allometric relationships for *E. caesia* sample 1.

**Supplemental Figure S8.** Allometric relationships for *E. caesia* sample 2.

**Supplemental Figure S9.** Allometric relationships for *E. caesia* sample 3.

**Supplemental Figure S10.** Allometric relationships for *E. caesia* sample 4.

**Supplemental Figure S11.** Allometric relationships for *E. caesia* sample 5.

**Supplemental Figure S12.** Allometric relationships for *E. diversicolor* sample 1.

**Supplemental Figure S13.** Allometric relationships for *E. diversicolor* sample 2.

**Supplemental Figure S14.** Allometric relationships for *E. diversicolor* sample 3.

**Supplemental Figure S15.** Allometric relationships for *E. diversicolor* sample 4.

**Supplemental Figure S16.** Allometric relationships for *E. incrassata* sample 1.

**Supplemental Figure S17.** Allometric relationships for *E. incrassata* sample 2.

**Supplemental Figure S18.** Allometric relationships for *E. incrassata* sample 3.

**Supplemental Figure S19.** Allometric relationships for *E. incrassata* sample 4.

**Supplemental Table S1.** Allometric covariation functions.

**Supplemental Table S2.** SMA regression statistics for allometric relationships for the 19 Eucalypt saplings.

**Supplemental Table S3.** SMA regression statistics for allometric relationships for the 4 Eucalypt species.

**Supplemental Table S4.** SMA regression statistics for the bivariate relationships depicted in Figure 4.

**Supplemental Note S1.** Incorporating conduit tapering into the model.

**Supplemental Note S2.** Extending model predictions to sub-trees and entire tree.

## Supplementary Material

kiac358_Supplementary_Data

## Notes

C.A.P. designed the research. C.A.P. and M.R developed the mathematical model. All authors performed the research, analyzed the data, and wrote the paper.

The author responsible for distribution of materials integral to the findings presented in this article in accordance with the policy described in the Instructions for Authors (https://academic.oup.com/plphys/pages/general-instructions) is: Charles A. Price (cprice9@utk.edu).
