# Supplementary material for: Flow similarity, stochastic branching, and quarter-power scaling in plants
Source: Plant Physiol. 2022 Aug 3;190(3):1854–65. doi: 10.1093/plphys/kiac358 (PMC9614476; doi:10.1093/plphys/kiac358)
Supplement: kiac358_Supplementary_Data [file kiac358_supplementary_data.zip › PP2022RA00476R1_Supplemental_Notes.pdf]

Supplemental Note 1

Our basic model assumes area-preserving branching and flow similarity (conservation of volumetric flow rate with branching), which together imply constant flow velocity through the branching structure. We also assume a constant pressure drop and a linear relationship between internal and external branching characteristics (eg. branching is area-preserving both for external branching structure and for internal branching conduits), and show that this leads to a prediction of surface-area scaling with volume to the power ¾. We then show that this prediction to be well-supported by empirical data.

Here we show that our model, with its basic assumptions of area-preserving external branching and flow similarity, is still consistent with both the data-supported prediction of surface-area scaling with volume to the power ¾ and the conduit dimensions tapering modelled by Savage et al. (2010) if the pressure drop varies in a certain way from parent to daughter branches.

Savage et al. (2010) show that theoretical optimality arguments lead to

$$r_{int\,k} \propto r_{ext\,k}^{\frac{1}{3}} \qquad (1)$$

and

$$N_{int\,k} \propto r_{ext\,k}^{\frac{4}{3}} \qquad (2)$$

where $r_{int\,k}$ and $r_{ext\,k}$ are the radii of the internal conduits and external branches at the kth branching generation and $N_{int\,k}$ is number of xylem conduits at level k.

If we also assume area-preserving bifurcating external branching

$$r_{ext\,k} \propto \sqrt{2}\, r_{ext\,k+1} = 2^{\frac{1}{2}}\, r_{ext\,k+1} \qquad (3)$$

where k+1 is the order of branching more distal than k, as per the usual labelling, then it follows that

$$r_{int\,k} = 2^{\frac{1}{6}}\, r_{int\,k+1} \qquad (4)$$

and

$$N_{int\,k} = 2^{\frac{2}{3}}\, N_{int\,k+1} \qquad (5)$$

If we then assume the key relationship we obtain from our basic model, which is the key result leading to the other relationships supported by the empirical data

$$l_{int\,k} = 2\, l_{int\,k+1} \qquad (6)$$

and assume, like Savage et al. (2010), that internal lengths mirror external lengths,

$$l_{ext\,k} = 2\, l_{ext\,k+1} \qquad (7)$$

then the total volumetric flow at order k

$$TQ_k = N_{int\,k} \frac{\pi {r_{int\,k}}^4 |\Delta P_k|}{8\eta l_{int\,k}}$$

$$= 2^{\frac{2}{3}}\, N_{int\,k+1} \frac{\pi {r_{int\,k+1}}^4 2^{\frac{2}{3}} |\Delta P_k|}{8\eta . 2 l_{int\,k}} \qquad \text{(from 4, 5 ,6)}$$

$$= 2^{\frac{1}{3}}\, N_{int\,k+1} \frac{\pi {r_{int\,k+1}}^4 |\Delta P_k|}{8\eta . l_{int\,k}} \qquad (8)$$

and if volumetric flow is conserved across generations then

$$TQ_{k+1} = N_{int\,k+1} \frac{\pi {r_{int\,k+1}}^4 |\Delta P_{k+1}|}{8\eta l_{int\,k+1}} = TQ_k = 2^{\frac{1}{3}}\, N_{int\,k+1} \frac{\pi {r_{int\,k+1}}^4 |\Delta P_k|}{8\eta . l_{int\,k}}$$

and thus

$$|\Delta P_{k+1}| = 2^{\frac{1}{3}} |\Delta P_k| \qquad (9)$$

or as a pressure gradient instead of a pressure drop

$$\frac{|\Delta P_{k+1}|}{l_{int\,k+1}} = \frac{2^{\frac{1}{3}}|\Delta P_k|}{2^{-1}\, l_{int\,k}} = 2^{\frac{4}{3}} \frac{|\Delta P_k|}{l_{int\,k}} \qquad (10)$$

This means that the assumptions of area-preserving external branching and flow similarity can be compatible with both the conduit tapering modelled in Savage et al. 2010 and the length to radius squared and surface area to volume to ¾ scalings that follow from (3) and (7) and find support in our empirical data, if pressure varies within the branching structure according to (9) and (10). Note this represents a steeper pressure drop/gradient than the original assumptions without tapering where pressure drop is constant, and thus pressure gradient doubles with each successive branching order as length halves (6).

Supplemental Note 2

Here we show that for a symmetric bifurcating tree structure:

1. length scales with radius squared through the whole structure at the scale of individual internodes
2. surface area scales with volume to the power ¾ through the whole structure at the scale of individual internodes
3. the total surface area of the tree scales with the total volume of the tree to the power ¾ as the tree increases in size

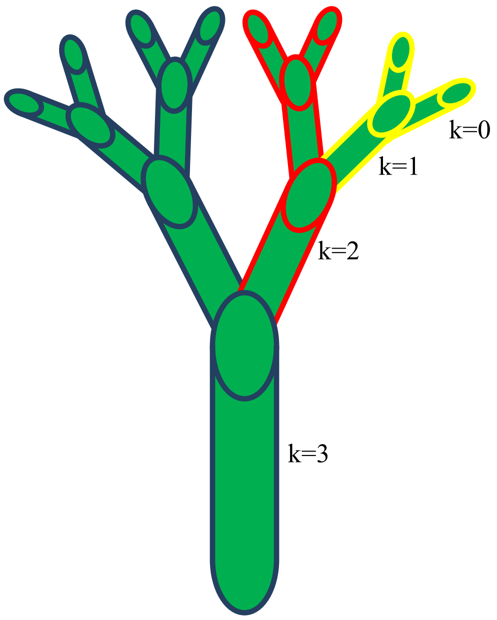

4. the total length of the tree scales with the basal radius of the tree squared as the tree increases in size

Let v_k, s_k, l_k, and r_k be the volume, surface area, length and radius of an internode at the kth level within a symmetric bifurcating tree structure, where the indexing of orders goes from the tips (order 0) to the base (order N), as shown in the figure i.e. the opposite direction to the normal labelling used in other parts of this paper. Let V_k, S_k, L_k, and R_k then be the total volume, surface area, length and radius of all internodes at the kth level within the structure, and TV_k, TS_k, and TL_k be the total volume, surface area and length of a subtree of order k ie the total volume (surface area/length) of a kth internode and all internodes distal to that internode.

If we assume $Q = \frac{\pi r^4 |\Delta P|}{8\eta l}$

then $l = \frac{Cr^4}{Q}$

where C=$\frac{\pi|\Delta P|}{8\eta}$.

If we also assume that branching is area preserving, that is $r_k=\sqrt{2}r_{k-1}$, and viscosity and pressure drop are the same in adjacent internodes as previously discussed, then we can establish the following relationships between branches at the $k-1^{th}$ and k^{th} levels

$$l_{k-1} = \frac{Cr_{k-1}^4}{Q_{k-1}} = \frac{C(\frac{1}{\sqrt{2}}r_k)^4}{\frac{1}{2}Q_k} = \frac{1}{2}\frac{Cr_k^4}{Q_k} = \frac{1}{2}l_k \quad (1)$$

and therefore

$$\frac{l_k}{r_k^2} = \frac{(\frac{1}{2})^k l_0}{\left(\left(\frac{1}{\sqrt{2}}\right)^k r_0\right)^2} = \frac{l_0}{r_0^2} \tag{2}$$

and so length scales with radius squared through the whole structure at the scale of individual internodes.

Then similarly for internode volumes

$$v_{k-1} = \pi r_{k-1}^2 l_{k-1} = \pi(\frac{1}{\sqrt{2}} r_k)^2 \frac{1}{2} l_k = \frac{1}{4}\pi r_k^2 l_k = \frac{1}{4} v_k \tag{3}$$

and surface areas

$$s_{k-1} = 2\pi r_{k-1} l_{k-1} = 2\pi \frac{1}{\sqrt{2}} r_k \frac{1}{2} l_k = \frac{1}{2\sqrt{2}} s_k = 2^{-\frac{3}{2}} s_k \tag{4}$$

And therefore, for any k,

$$\frac{v_{k-1}^{\frac{3}{4}}}{s_{k-1}} = \frac{\left(\frac{1}{4} v_k\right)^{\frac{3}{4}}}{\left(2^{-\frac{3}{2}} s_k\right)} = \frac{2^{-2\times\frac{3}{4}}}{2^{-\frac{3}{2}}} \frac{v_k^{\frac{3}{4}}}{s_k} = \frac{v_k^{\frac{3}{4}}}{s_k} \tag{5}$$

and so surface area scales with volume to the power ¾ through the whole structure at the scale of individual internodes.

From Eqn 3, and because there are twice as many branches at order k-1 compared to order k

$$V_{k-1} = \frac{1}{2} V_k \tag{6}$$

Similarly, Eqn 4 says that,

$$s_{k+1} = 2\sqrt{2} s_k$$

and because there are twice as many branches at order k-1 compared to order k

$$S_{k-1} = \frac{1}{\sqrt{2}} S_k \qquad (7)$$

It follows from Eqn 6 that

$$TV_k = V_k + V_{k-1} + V_{k-2} + \cdots + V_1 + V_0$$

$$= V_k + \frac{1}{2} V_k + \left(\frac{1}{2}\right)^2 V_k + \cdots + \left(\frac{1}{2}\right)^{k-1} V_k + \left(\frac{1}{2}\right)^k V_k$$

$$= V_k \left(\frac{1-\left(\frac{1}{2}\right)^k}{1-\frac{1}{2}}\right) = 2V_k \left(1 - \left(\frac{1}{2}\right)^k\right) = 2v_k \left(1 - \left(\frac{1}{2}\right)^k\right) \qquad (8)$$

and from Eqn 7 that

$$TS_k = S_k + S_{k-1} + S_{k-2} + \cdots + S_1 + S_0$$

$$= S_k + \frac{1}{\sqrt{2}} S_k + \left(\frac{1}{\sqrt{2}}\right)^2 S_k + \cdots + \left(\frac{1}{\sqrt{2}}\right)^{k-1} S_k + \left(\frac{1}{\sqrt{2}}\right)^k S_k$$

$$= S_k \left(\frac{1-\left(\frac{1}{\sqrt{2}}\right)^k}{1-\frac{1}{\sqrt{2}}}\right) = s_k \left(\frac{1-\left(\frac{1}{\sqrt{2}}\right)^k}{1-\frac{1}{\sqrt{2}}}\right) \qquad (9)$$

We then consider the proportional difference in total volume between a tree of order k+1 and another of order k.

$$\frac{TV_{k+1}}{TV_k} = \frac{2TV_k + 4v_k}{TV_k} = 2 + \frac{4v_k}{2v_k(1 - {1}/{2}^k)}$$

$$= 2 + \frac{2}{\left(1-\left(\frac{1}{2}\right)^{k}\right)} \qquad (10)$$

which tends towards 4 as k becomes large.

We similarly consider the proportional difference in total surface area between a tree of order k+1 and another of order k.

$$\frac{TS_{k+1}}{TS_k} = \frac{2TS_k + 2\sqrt{2}s_k}{TS_k} = 2 + \frac{2\sqrt{2}s_k}{s_k\left[\frac{1-{1/\sqrt{2}}^{k}}{1-{1/\sqrt{2}}}\right]}$$

$$= 2 + \frac{2\sqrt{2}\left(1-\frac{1}{\sqrt{2}}\right)}{1-\left(\frac{1}{\sqrt{2}}\right)^{k}}$$

which tends towards

$$2 + 2\sqrt{2}\left(1-\frac{1}{\sqrt{2}}\right) = 2\sqrt{2}$$

as k becomes large.

And so as k becomes large

$$\frac{\log\left(\frac{TV_{k+1}}{TV_k}\right)}{\log\left(\frac{TS_{k+1}}{TS_k}\right)} \to \frac{\log 4}{\log 2\sqrt{2}} = \frac{2\log 2}{{3/2}\log 2} = \frac{4}{3} \qquad (11)$$

which means that the total volume of the tree scales with the total surface area of the tree to the power 4/3 as the tree increases in size, or in other words the total surface area of the tree scales with the total volume of the tree to the power ¾ as the tree increases in size.

Furthermore, from Eqn 1, combined with the fact that there are twice as many internodes at level k compared to level k-1

$$L_k = L_k \tag{12}$$

and so

$$TL_k = L_k + L_{k-1} + L_{k-2} + \cdots + L_1 + L_0$$

$$= L_k + L_k + L_k + \cdots + L_k + L_k$$

$$= kL_k = kl_k \tag{13}$$

We can then consider the proportional difference in total length between a tree of order k+1 and another of order k.

$$\frac{TL_{k+1}}{TL_k} = \frac{2TL_k + 2l_k}{TL_k} = 2 + \frac{2l_k}{kl_k} = 2 + \frac{2}{k} \tag{14}$$

which tends towards 2 as k becomes large.

And so as k becomes large, the scaling exponent between the total length of a tree and its basal radius

$$\frac{\log\left(\frac{TL_{k+1}}{TL_k}\right)}{\log\left(\frac{r_{k+1}}{r_k}\right)} \to \frac{\log(2)}{\log(\sqrt{2})} = 2 \tag{15}$$

which means that the total length of the tree scales with the basal radius of the tree squared as the tree increases in size.
