# Supplementary material for: Flow similarity, stochastic branching, and quarter-power scaling in plants
Source: Plant Physiol. 2022 Aug 3;190(3):1854–65. doi: 10.1093/plphys/kiac358 (PMC9614476; doi:10.1093/plphys/kiac358)
Supplement: kiac358_Supplementary_Data [file kiac358_supplementary_data.zip › PP2022RA00476R1_Supplemental_Table_1.pdf]

Supplemental Table S1. Allometric covariation

As shown in Table 1 of the main manuscript, the exponents for the scaling functions between length (L), diameter (D), surface area (SA) and volume (V), can all be expressed as a function of alpha (α), the exponent from the LvD relationship. For collections of allometric exponents, one can explore the functional relationships between the scaling exponents themselves. The table below contains predictions for the 15 pairwise covariation functions between the six scaling relationships.

Y variable	X variable	Covariation Function
$SA \propto V^{\frac{(\alpha+1)}{(\alpha+2)}}$	$L \propto D^{\alpha}$	$\frac{(\alpha+1)}{(\alpha+2)} = \frac{(\alpha+1)}{(\alpha+2)}$
$D \propto V^{\frac{1}{(\alpha+2)}}$	$L \propto D^{\alpha}$	$\frac{1}{(\alpha+2)} = \frac{1}{(\alpha+2)}$
$L \propto V^{\frac{\alpha}{(\alpha+2)}}$	$L \propto D^{\alpha}$	$\frac{\alpha}{(\alpha+2)} = \frac{\alpha}{(\alpha+2)}$
$D \propto SA^{\frac{1}{(\alpha+1)}}$	$L \propto D^{\alpha}$	$\frac{1}{(\alpha+1)} = \frac{1}{(\alpha+1)}$
$L \propto SA^{\frac{\alpha}{(\alpha+1)}}$	$L \propto D^{\alpha}$	$\frac{\alpha}{(\alpha+1)} = \frac{\alpha}{(\alpha+1)}$
$D \propto V^{\frac{1}{(\alpha+2)}}$	$SA \propto V^{\frac{(\alpha+1)}{(\alpha+2)}}$	$\frac{1}{\alpha+2} = 1 - \left(\frac{\alpha+1}{\alpha+2}\right)$
$L \propto V^{\frac{\alpha}{(\alpha+2)}}$	$SA \propto V^{\frac{(\alpha+1)}{(\alpha+2)}}$	$\frac{\alpha}{\alpha+2} = 2\left(\frac{\alpha+1}{\alpha+2}\right) - 1$
$D \propto SA^{\frac{1}{(\alpha+1)}}$	$SA \propto V^{\frac{(\alpha+1)}{(\alpha+2)}}$	$\frac{1}{(\alpha+1)} = \frac{\alpha+2}{\alpha+1} - 1$
$L \propto SA^{\frac{\alpha}{(\alpha+1)}}$	$SA \propto V^{\frac{(\alpha+1)}{(\alpha+2)}}$	$\frac{\alpha}{\alpha+1} = \frac{\alpha^3+2\alpha^2}{\alpha^3+3\alpha^2+2\alpha}$
$L \propto V^{\frac{\alpha}{(\alpha+2)}}$	$D \propto V^{\frac{1}{(\alpha+2)}}$	$\frac{\alpha}{\alpha+2} = 1 - \left(\frac{2}{\alpha+2}\right)$
$D \propto SA^{\frac{1}{(\alpha+1)}}$	$D \propto V^{\frac{1}{(\alpha+2)}}$	$\frac{1}{(\alpha+1)} = \frac{1}{1-\left(\frac{1}{\alpha+2}\right)} - 1$
$L \propto SA^{\frac{\alpha}{(\alpha+1)}}$	$D \propto V^{\frac{1}{(\alpha+2)}}$	$\frac{\alpha}{\alpha+1} = \frac{1}{\left(\frac{1}{\alpha+2}\right)-1} + 2$
$D \propto SA^{\frac{1}{(\alpha+1)}}$	$L \propto V^{\frac{\alpha}{(\alpha+2)}}$	$\frac{1}{(\alpha+1)} = \frac{2}{\left(\frac{\alpha}{\alpha+2}\right)+1} - 1$
$L \propto SA^{\frac{\alpha}{(\alpha+1)}}$	$L \propto V^{\frac{\alpha}{(\alpha+2)}}$	$\frac{\alpha}{(\alpha+1)} = \frac{\alpha^2+2\alpha}{\alpha^2+3\alpha+2}$
$D \propto SA^{\frac{1}{(\alpha+1)}}$	$L \propto SA^{\frac{\alpha}{(\alpha+1)}}$	$\frac{1}{\alpha+1} = 1 - \left(\frac{\alpha}{\alpha+1}\right)$
