# Supplementary material for: Flow similarity, stochastic branching, and quarter-power scaling in plants
Source: Plant Physiol. 2022 Aug 3;190(3):1854–65. doi: 10.1093/plphys/kiac358 (PMC9614476; doi:10.1093/plphys/kiac358)
Supplement: kiac358_Supplementary_Data [file kiac358_supplementary_data.zip › Figures_S1_S19 w legends.pdf]

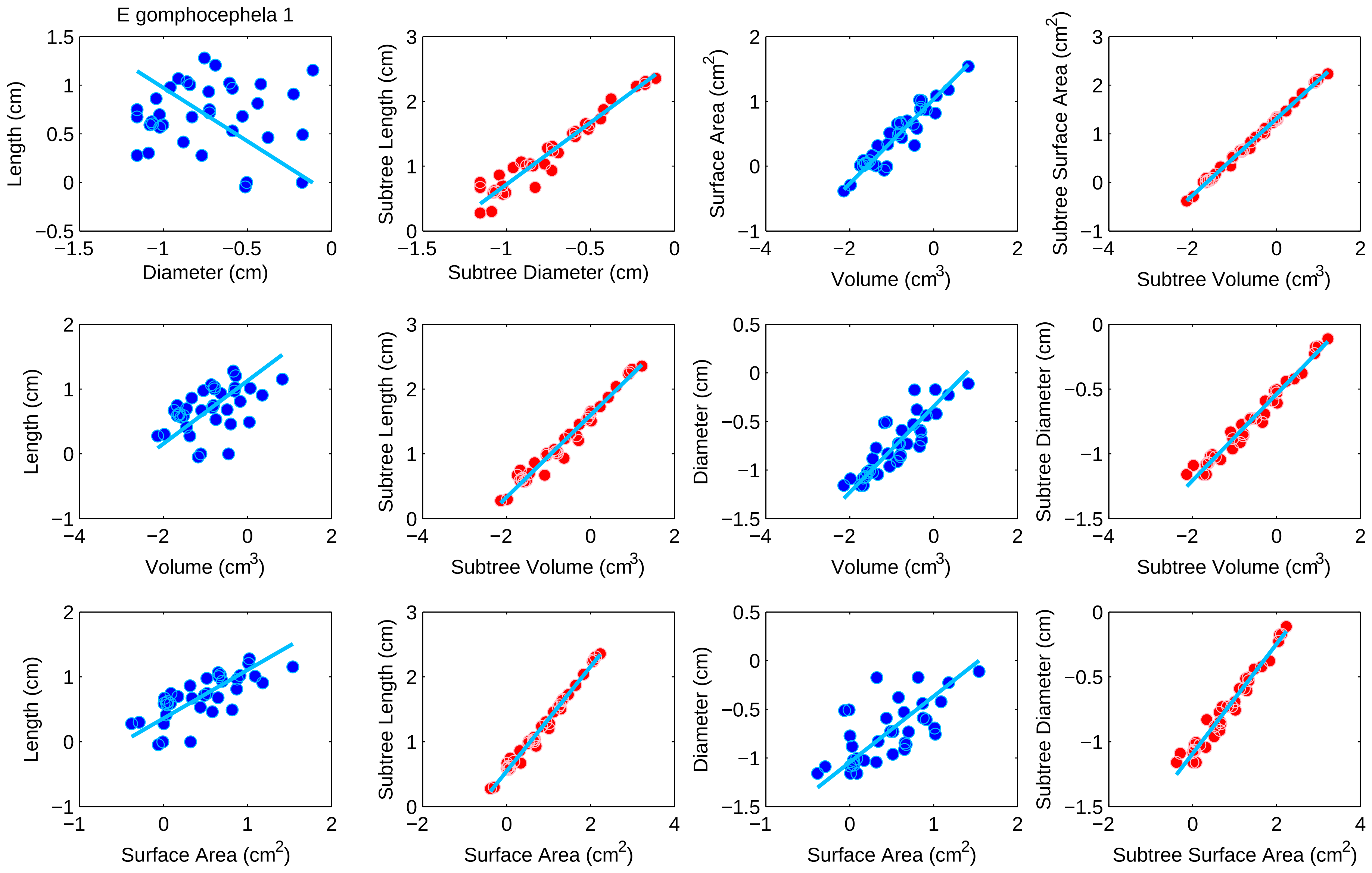

Figure S1. Allometric relationships for E. gomphocephela sample 1. Sapling dimensions for raw data (blue symbols) and subtrees (red symbols).

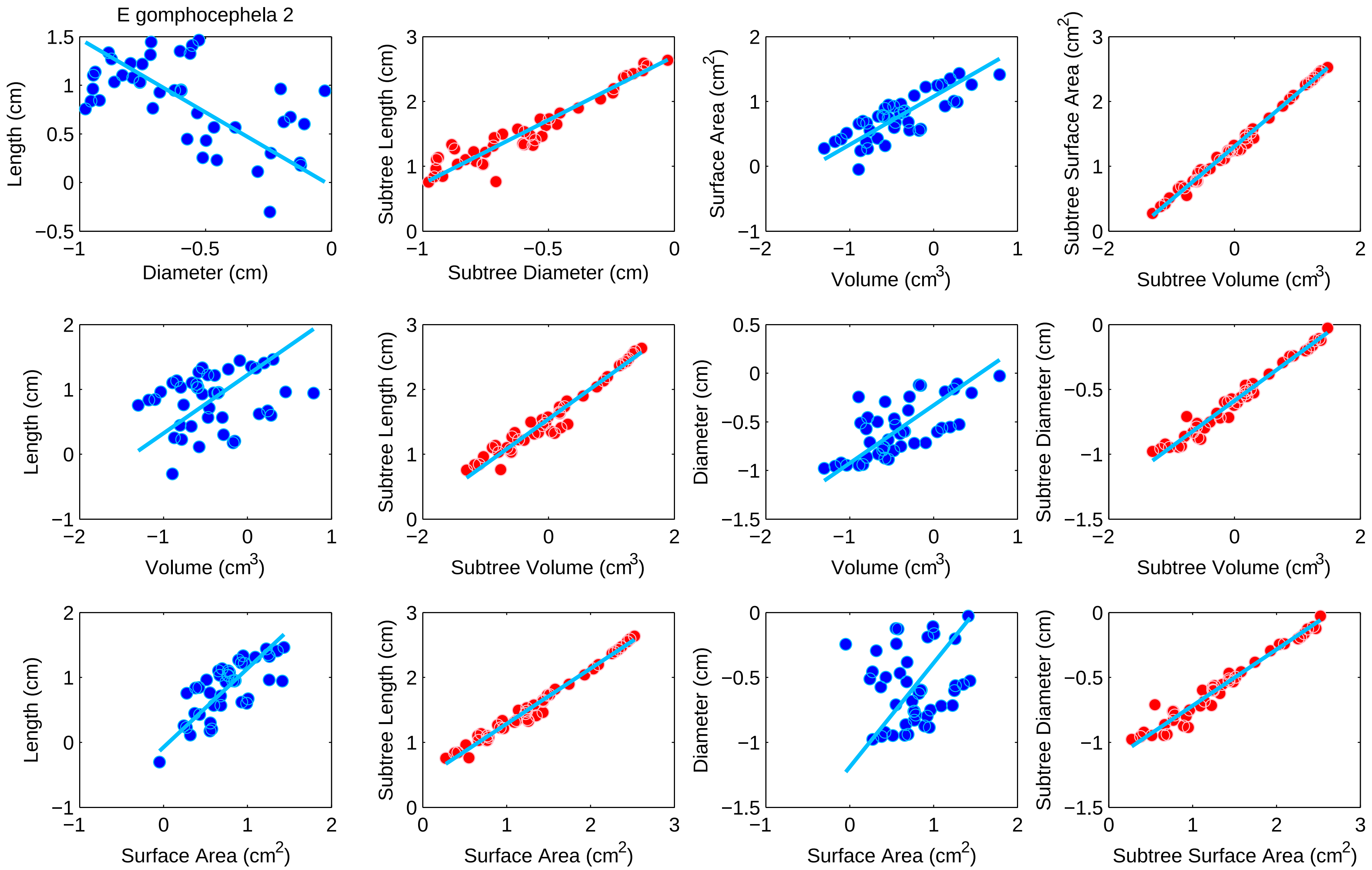

Figure S2. Allometric relationships for E. gomphocephela sample 2. Sapling dimensions for raw data (blue symbols) and subtrees (red symbols).

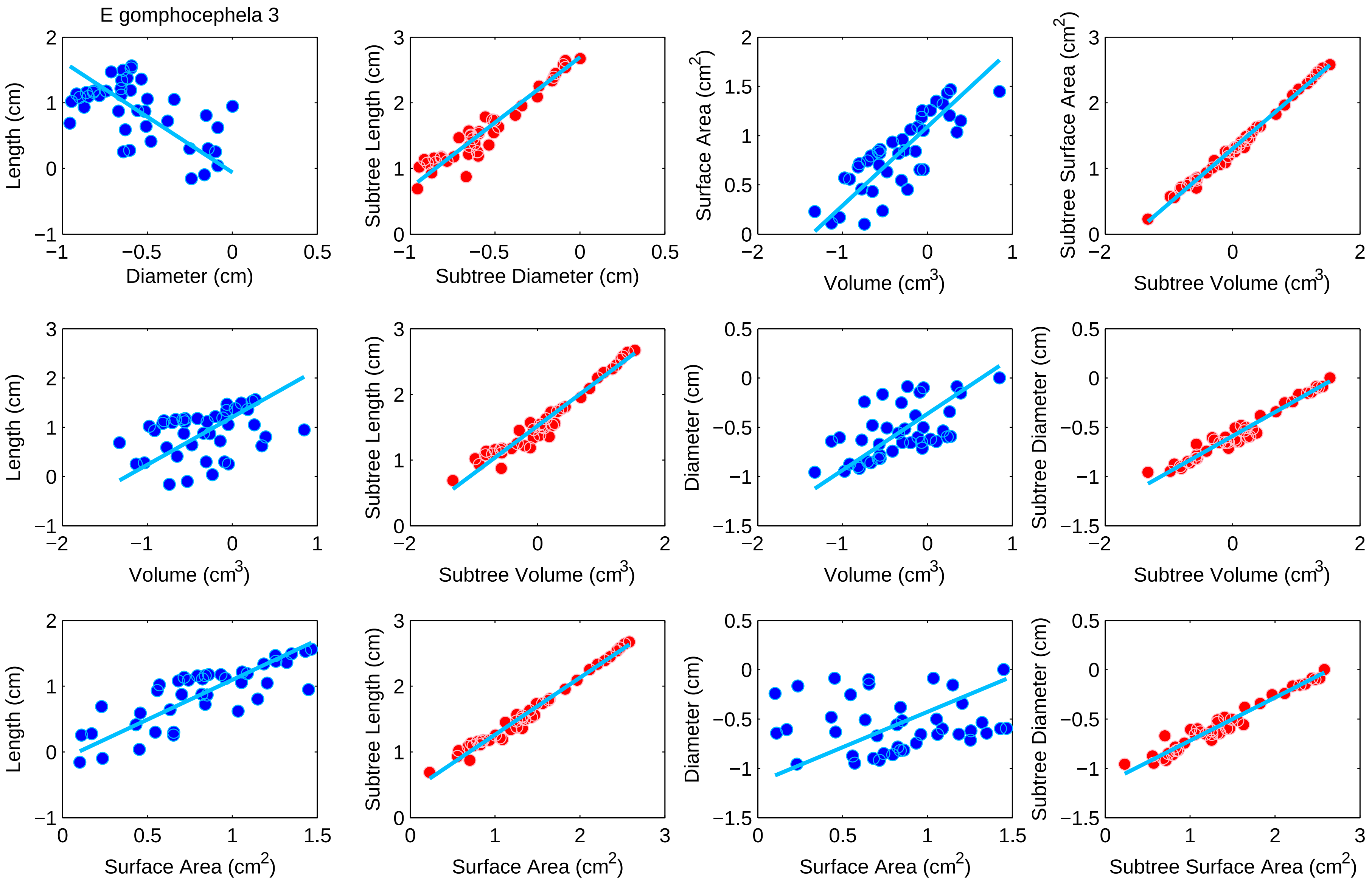

Figure S3. Allometric relationships for E. gomphocephela sample 3. Sapling dimensions for raw data (blue symbols) and subtrees (red symbols).

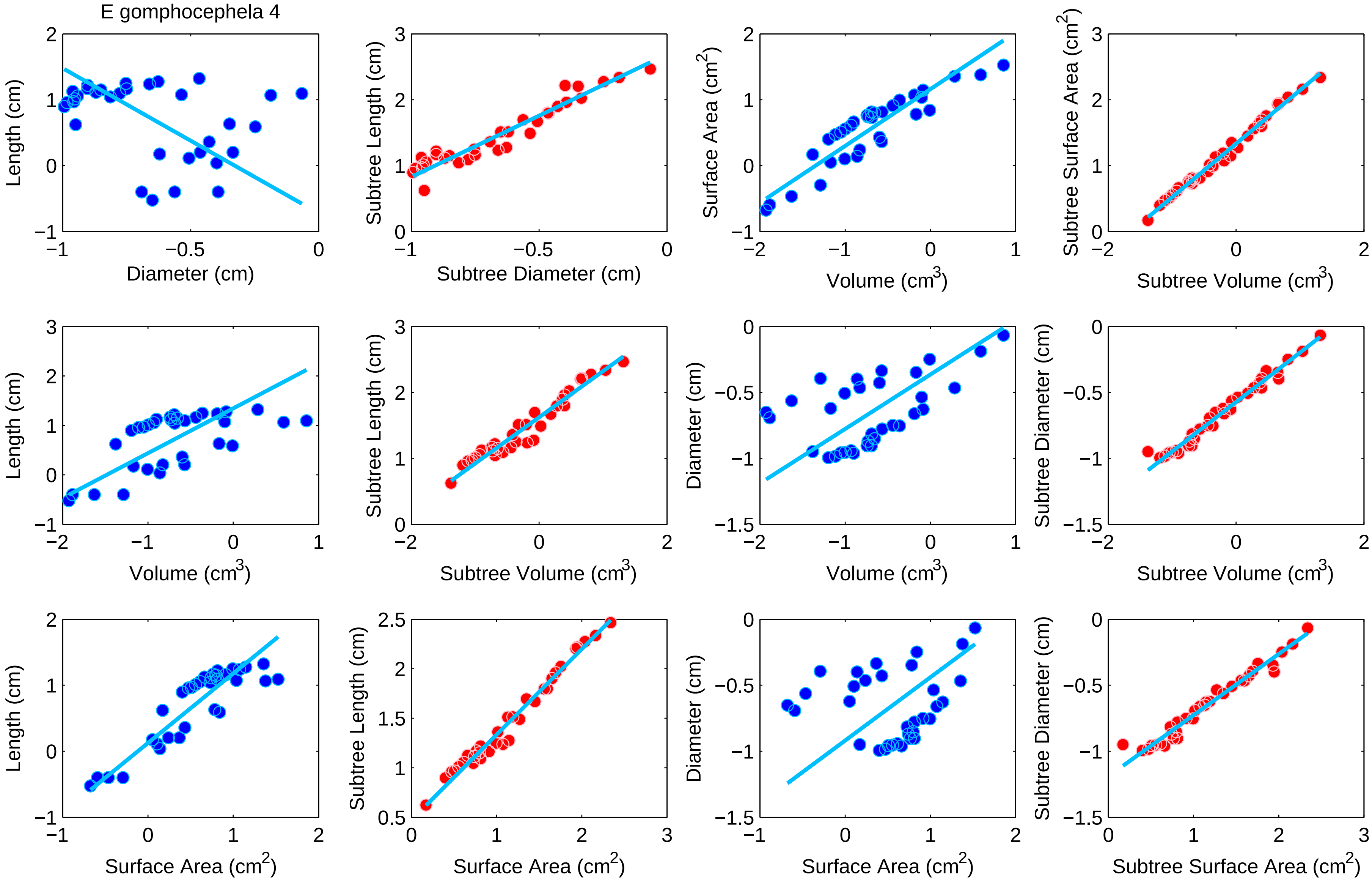

Figure S4. Allometric relationships for E. gomphocephela sample 4. Sapling dimensions for raw data (blue symbols) and subtrees (red symbols).

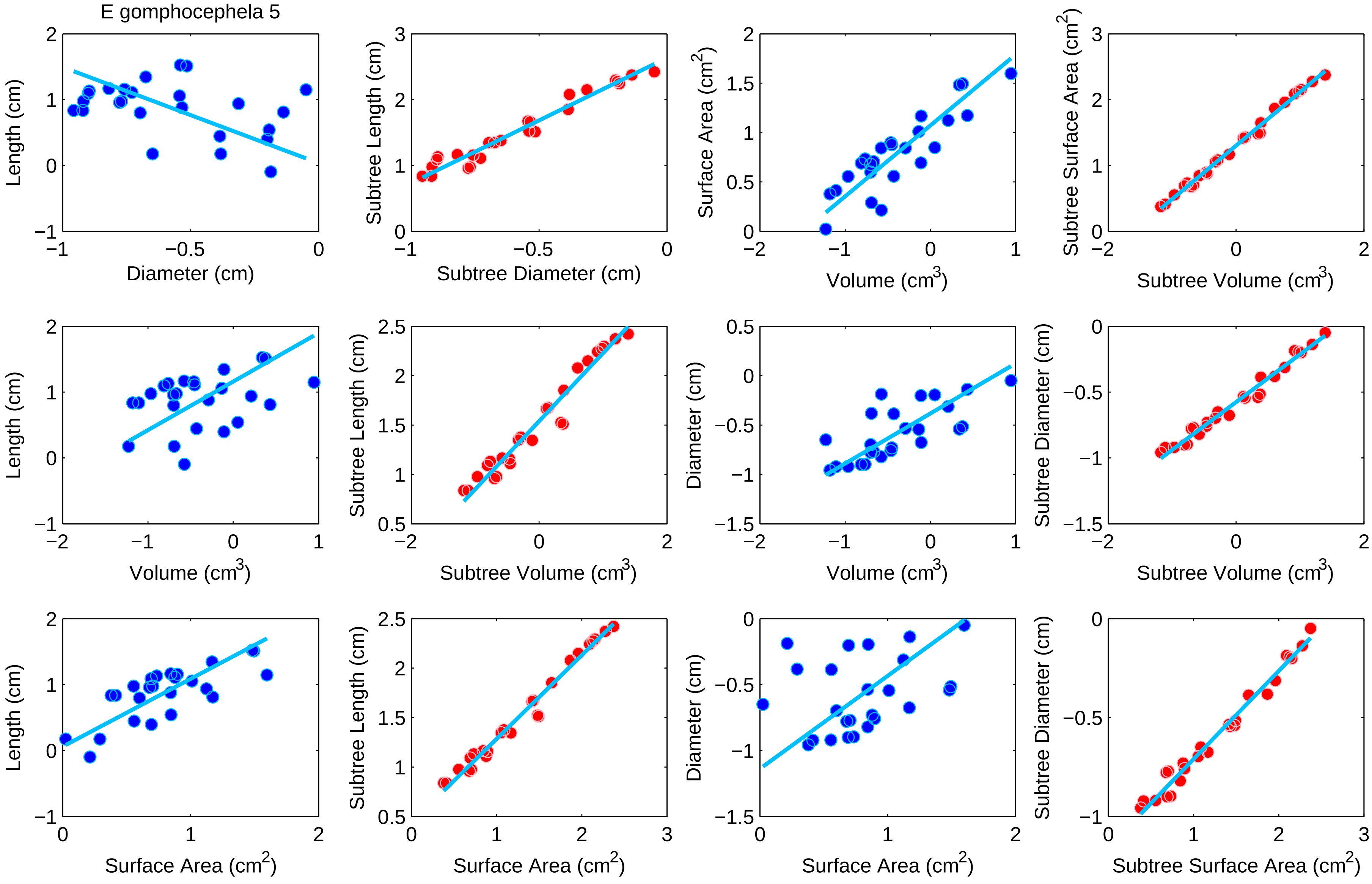

Figure S5. Allometric relationships for E. gomphocephela sample 5. Sapling dimensions for raw data (blue symbols) and subtrees (red symbols).

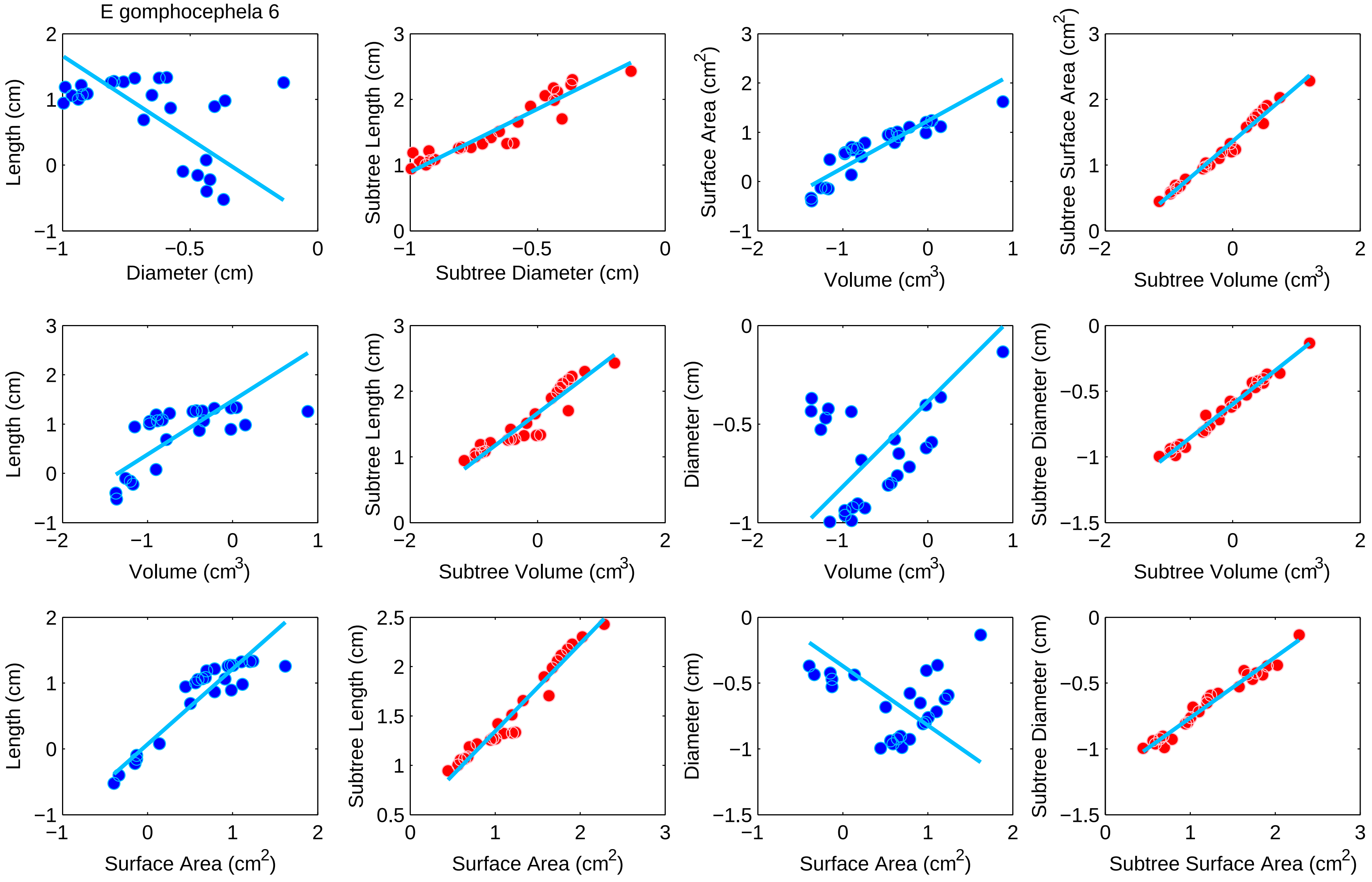

Figure S6. Allometric relationships for E. gomphocephela sample 6. Sapling dimensions for raw data (blue symbols) and subtrees (red symbols).

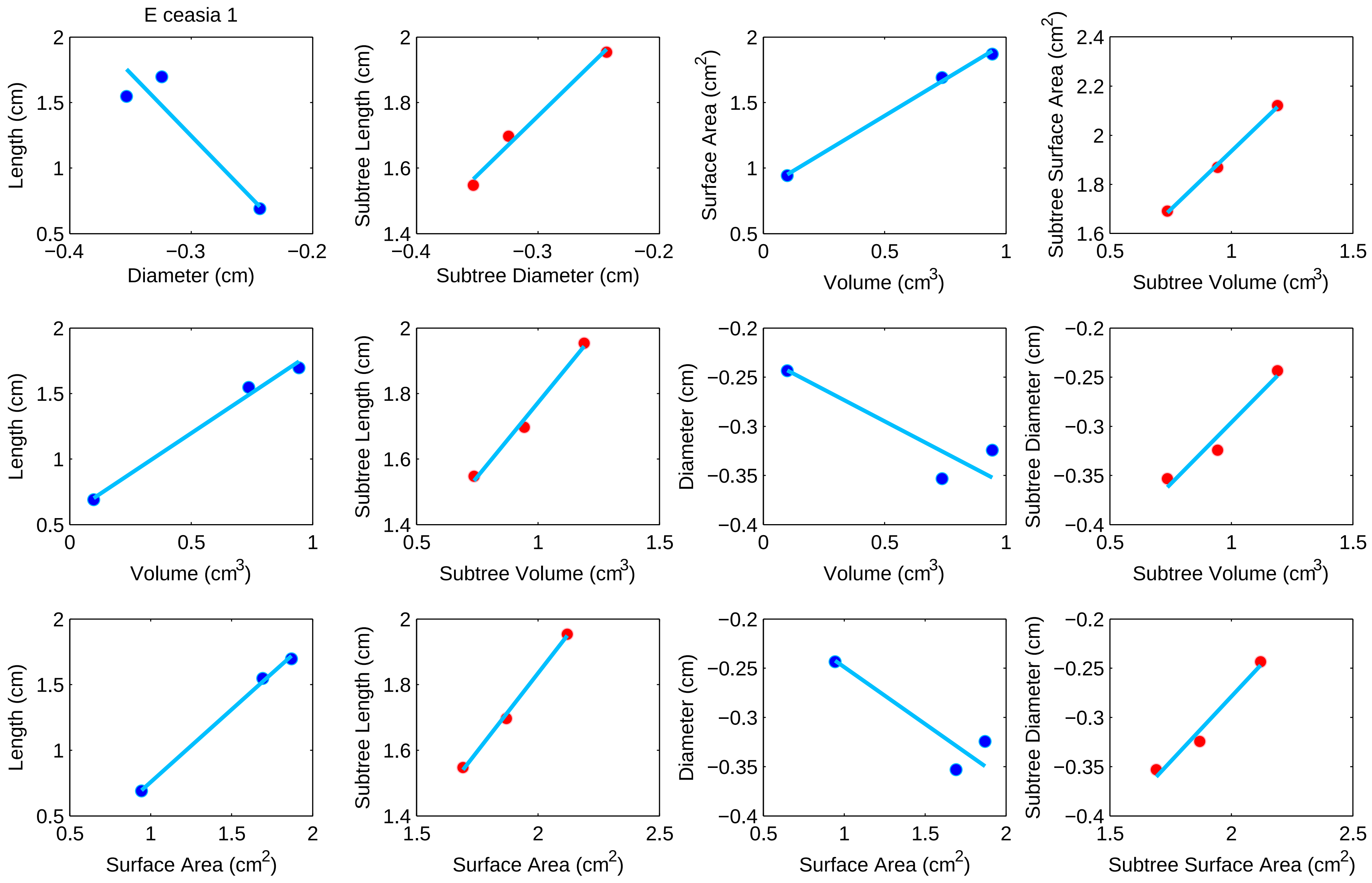

Figure S7. Allometric relationships for E. ceasia sample 1. Sapling dimensions for raw data (blue symbols) and subtrees (red symbols).

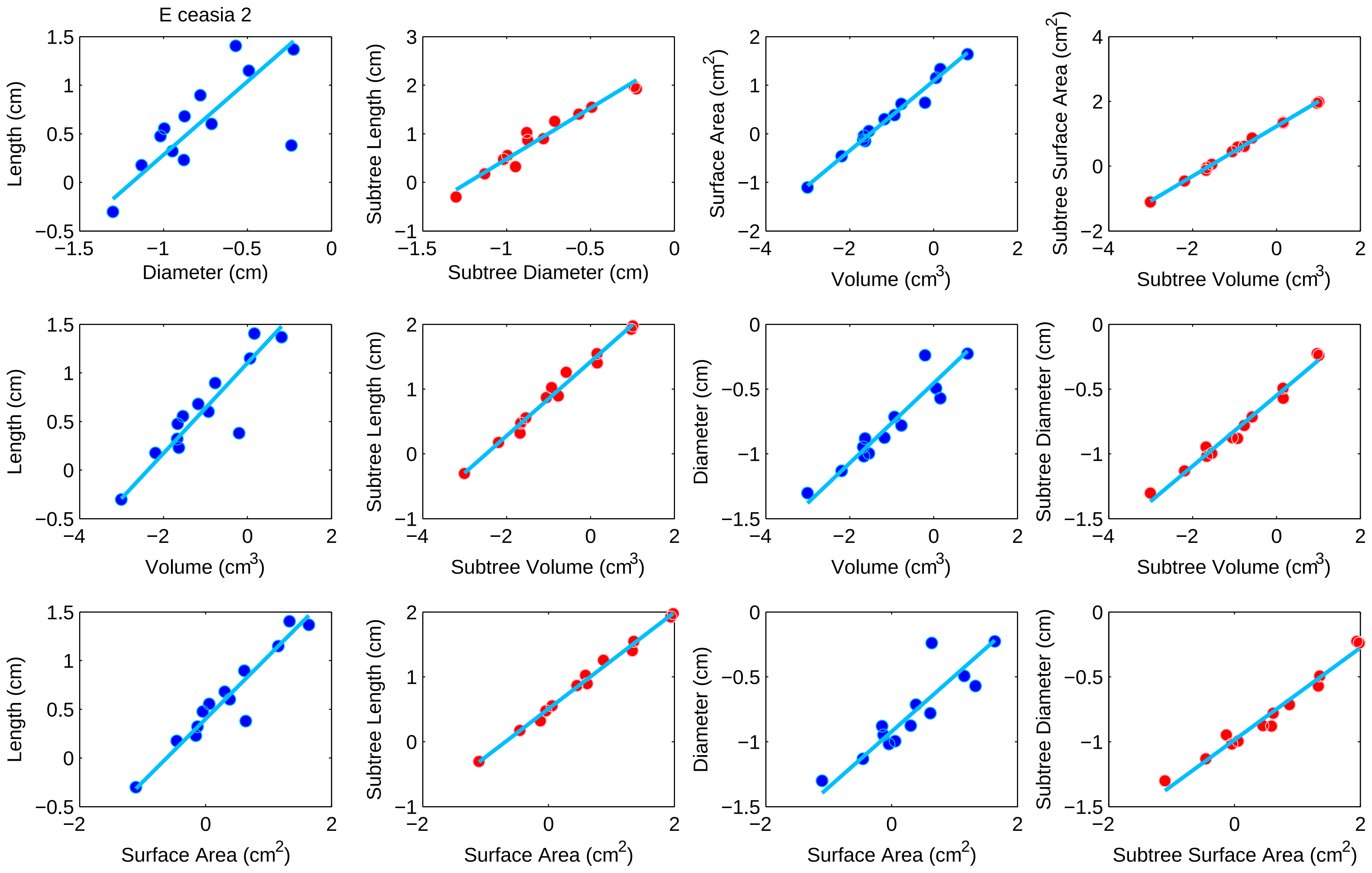

Figure S8. Allometric relationships for E. ceasia sample 2. Sapling dimensions for raw data (blue symbols) and subtrees (red symbols).

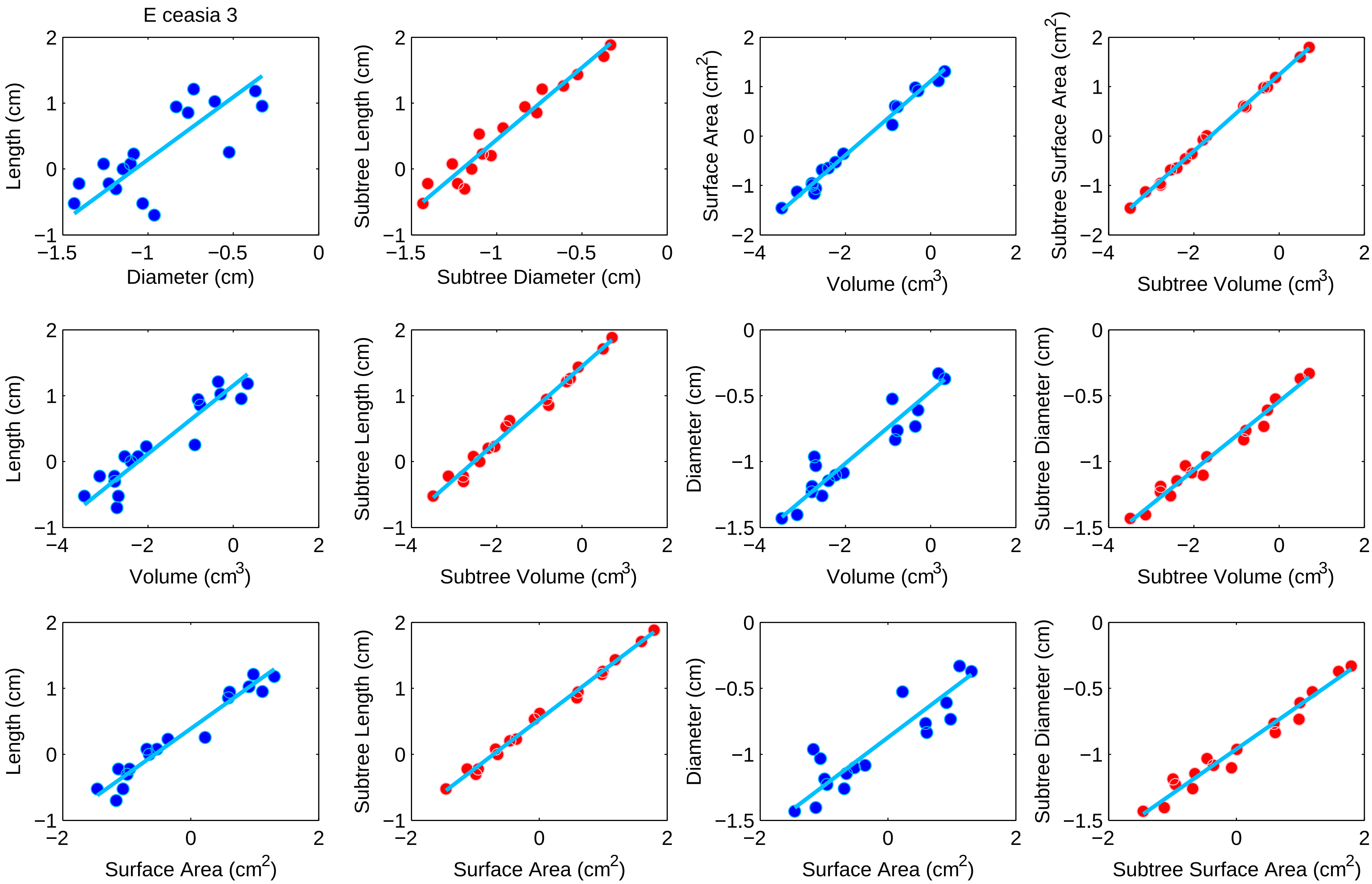

Figure S9. Allometric relationships for E. ceasia sample 3. Sapling dimensions for raw data (blue symbols) and subtrees (red symbols).

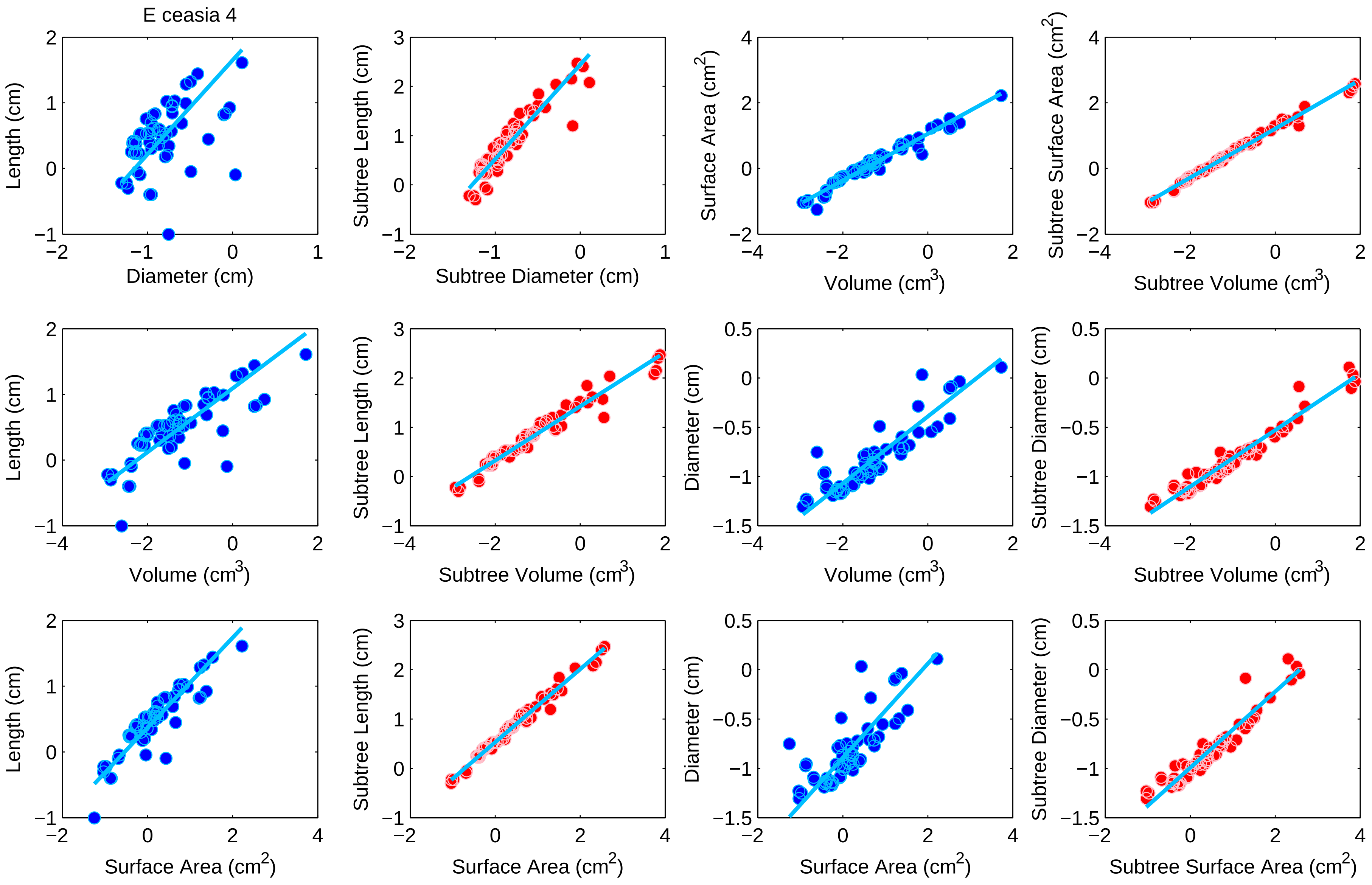

Figure S10. Allometric relationships for E. ceasia sample 4. Sapling dimensions for raw data (blue symbols) and subtrees (red symbols).

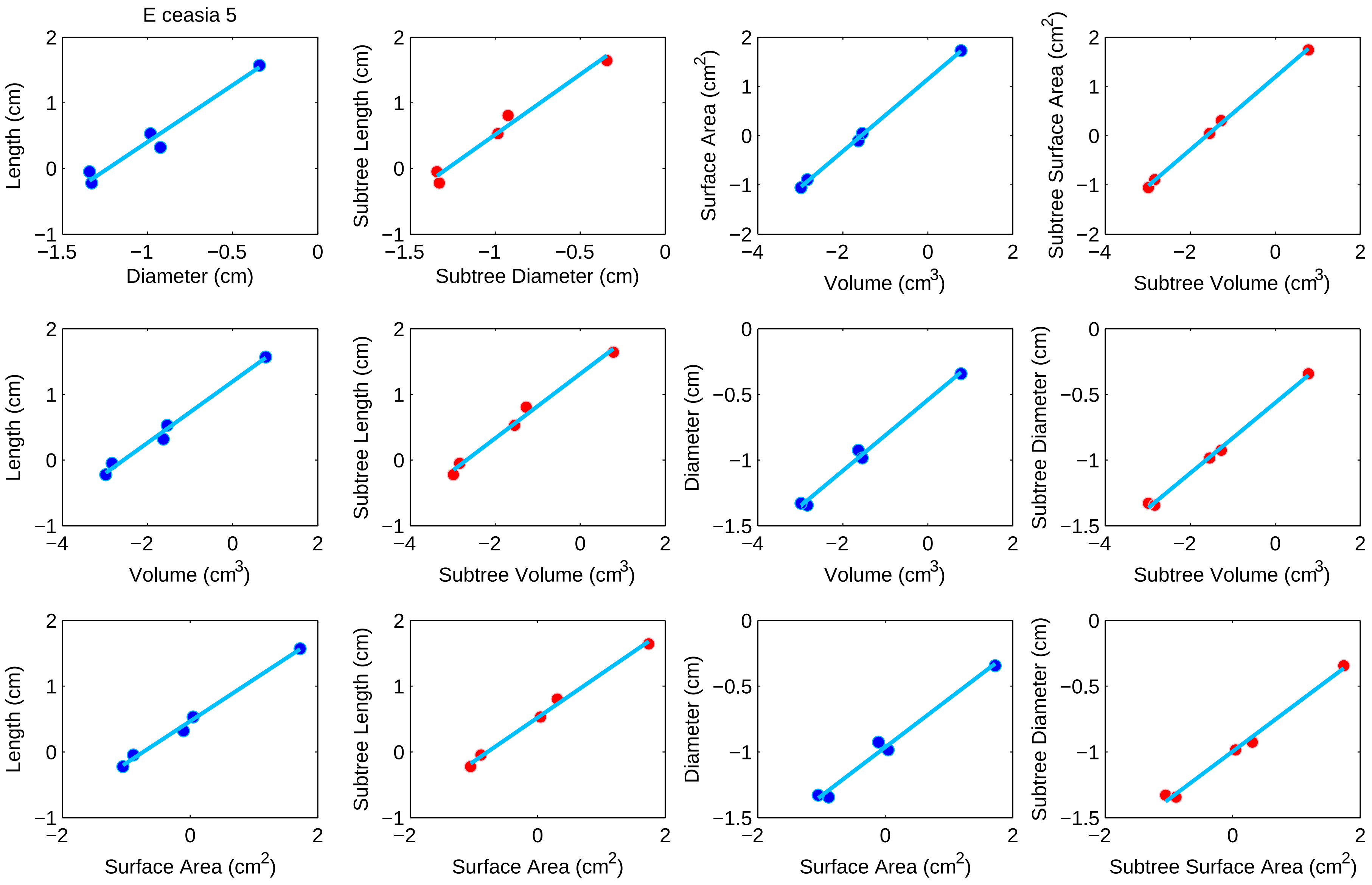

Figure S11. Allometric relationships for E. ceasia sample 5. Sapling dimensions for raw data (blue symbols) and subtrees (red symbols).

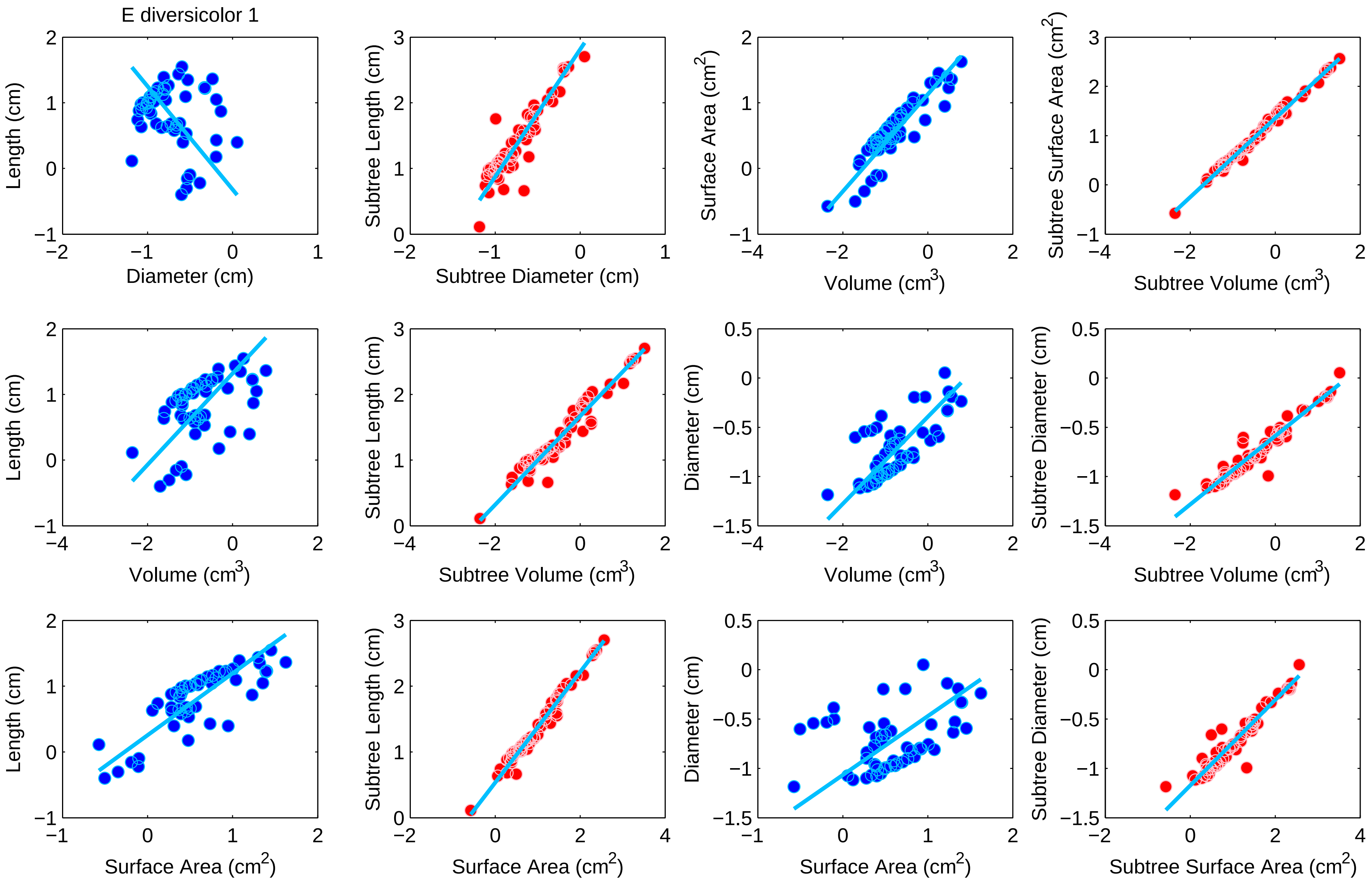

Figure S12. Allometric relationships for E. diversicolor sample 1. Sapling dimensions for raw data (blue symbols) and subtrees (red symbols).

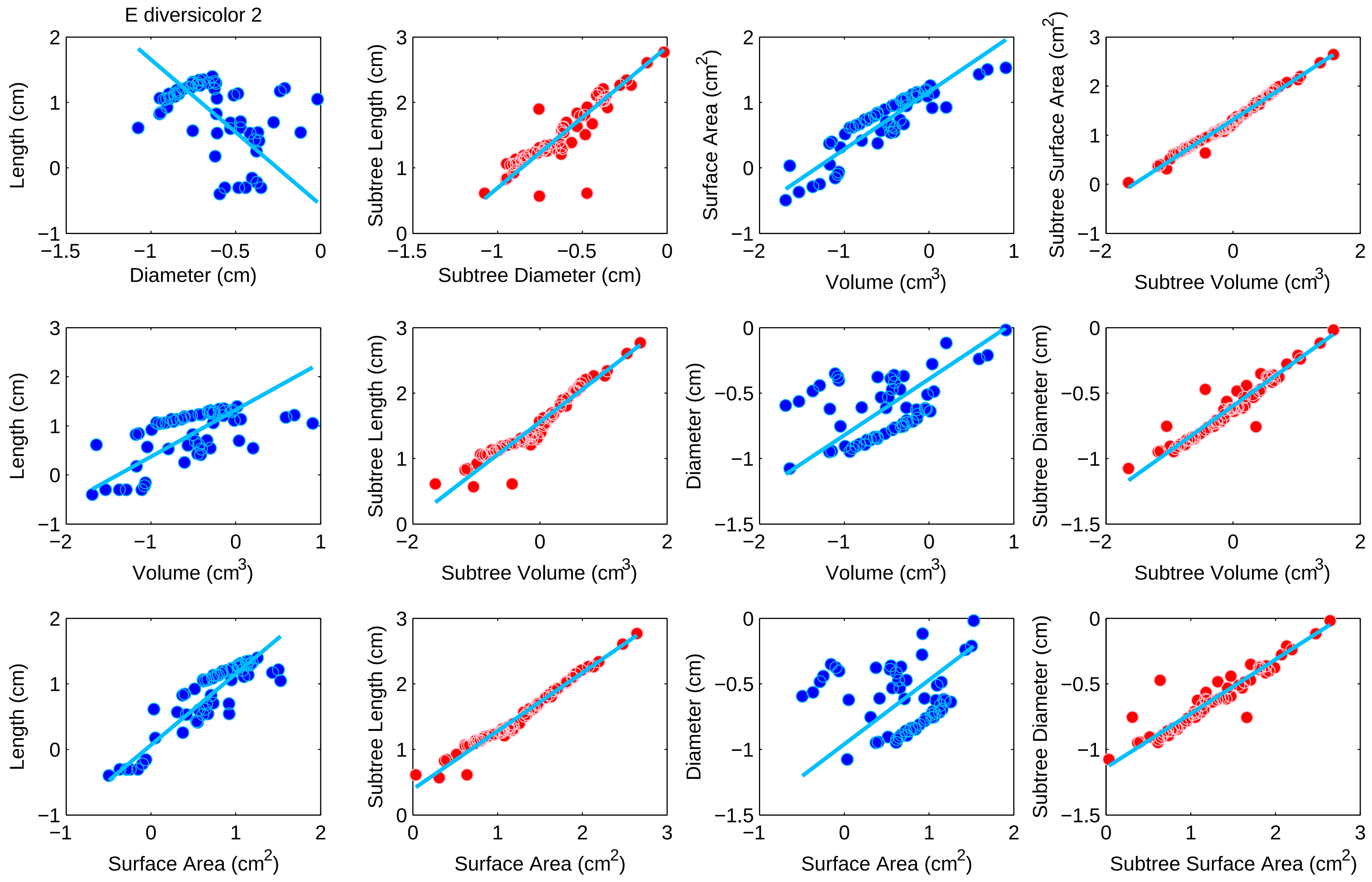

Figure S13. Allometric relationships for E. diversicolor sample 2. Sapling dimensions for raw data (blue symbols) and subtrees (red symbols).

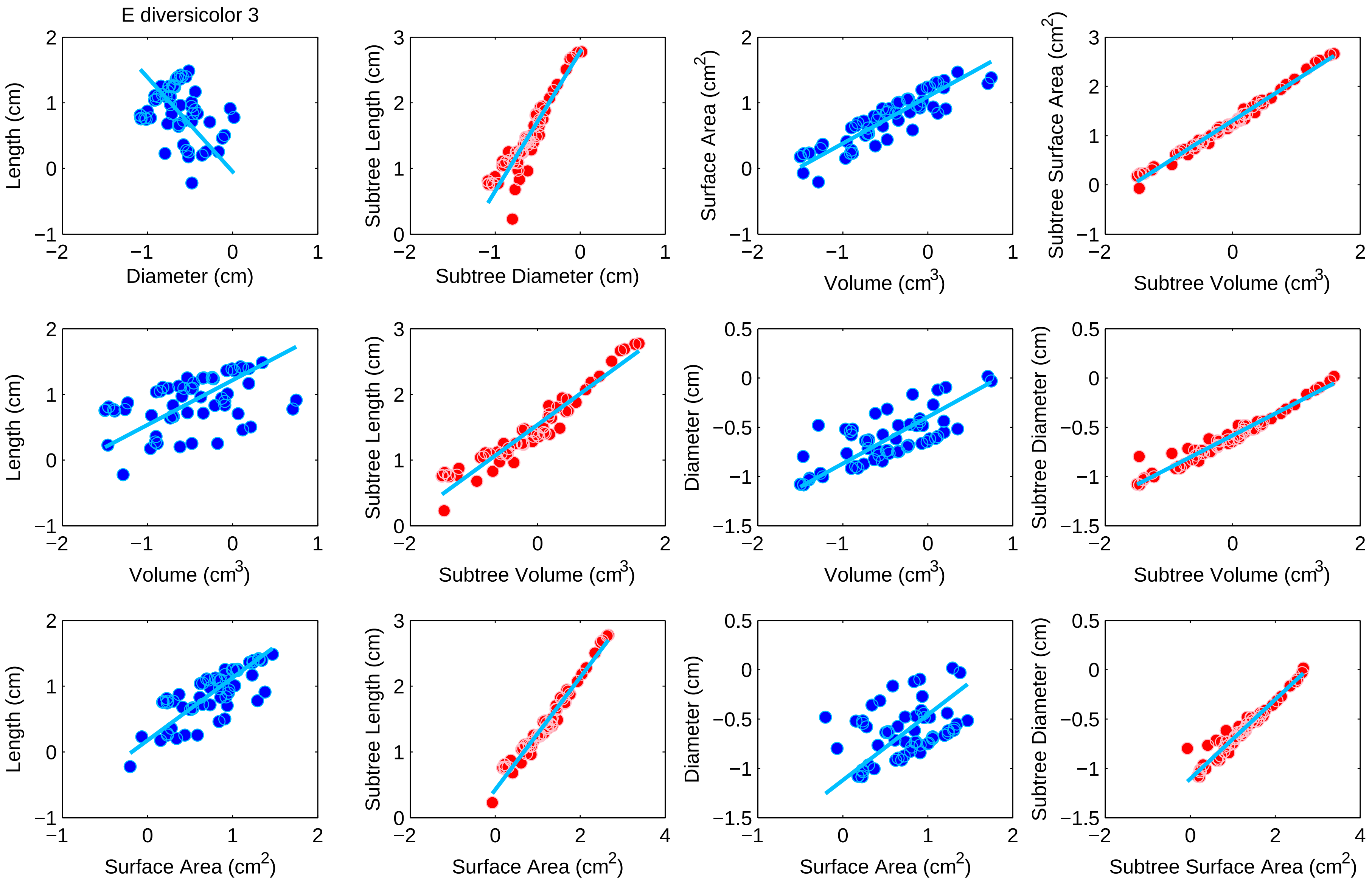

Figure S14. Allometric relationships for E. diversicolor sample 3. Sapling dimensions for raw data (blue symbols) and subtrees (red symbols).

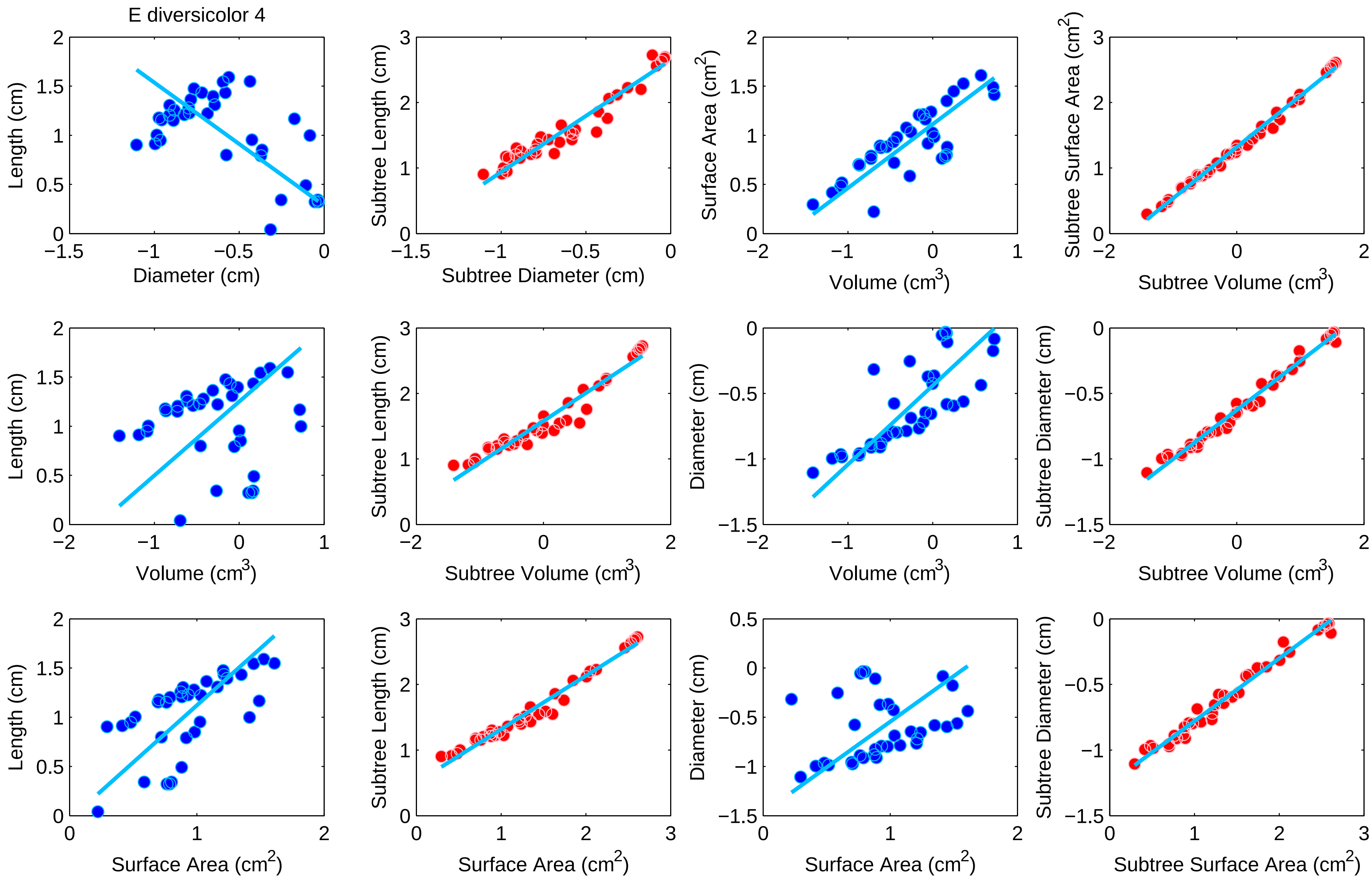

Figure S15. Allometric relationships for E. diversicolor sample 4. Sapling dimensions for raw data (blue symbols) and subtrees (red symbols).

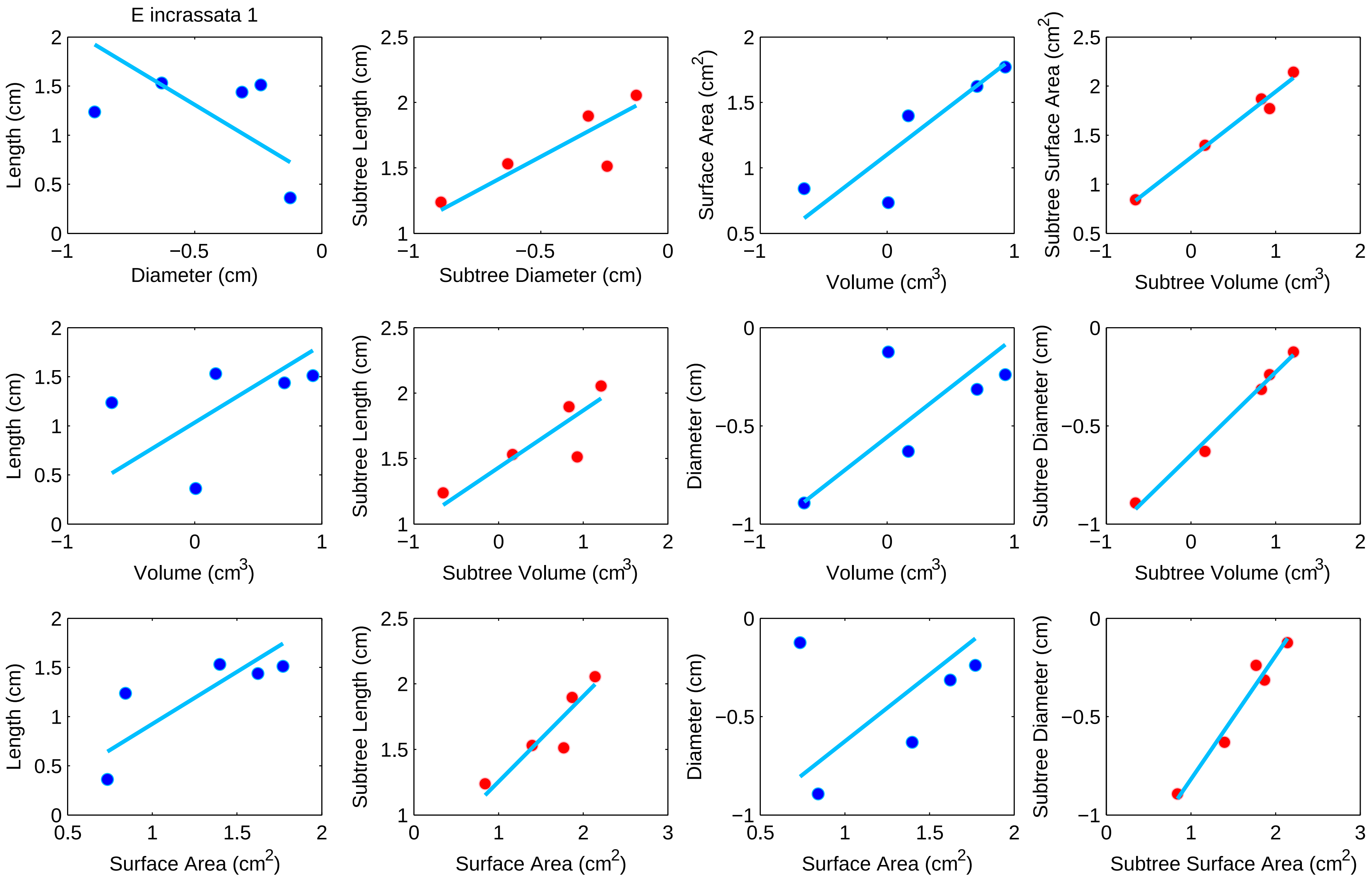

Figure S16. Allometric relationships for E. incrassata sample 1. Sapling dimensions for raw data (blue symbols) and subtrees (red symbols).

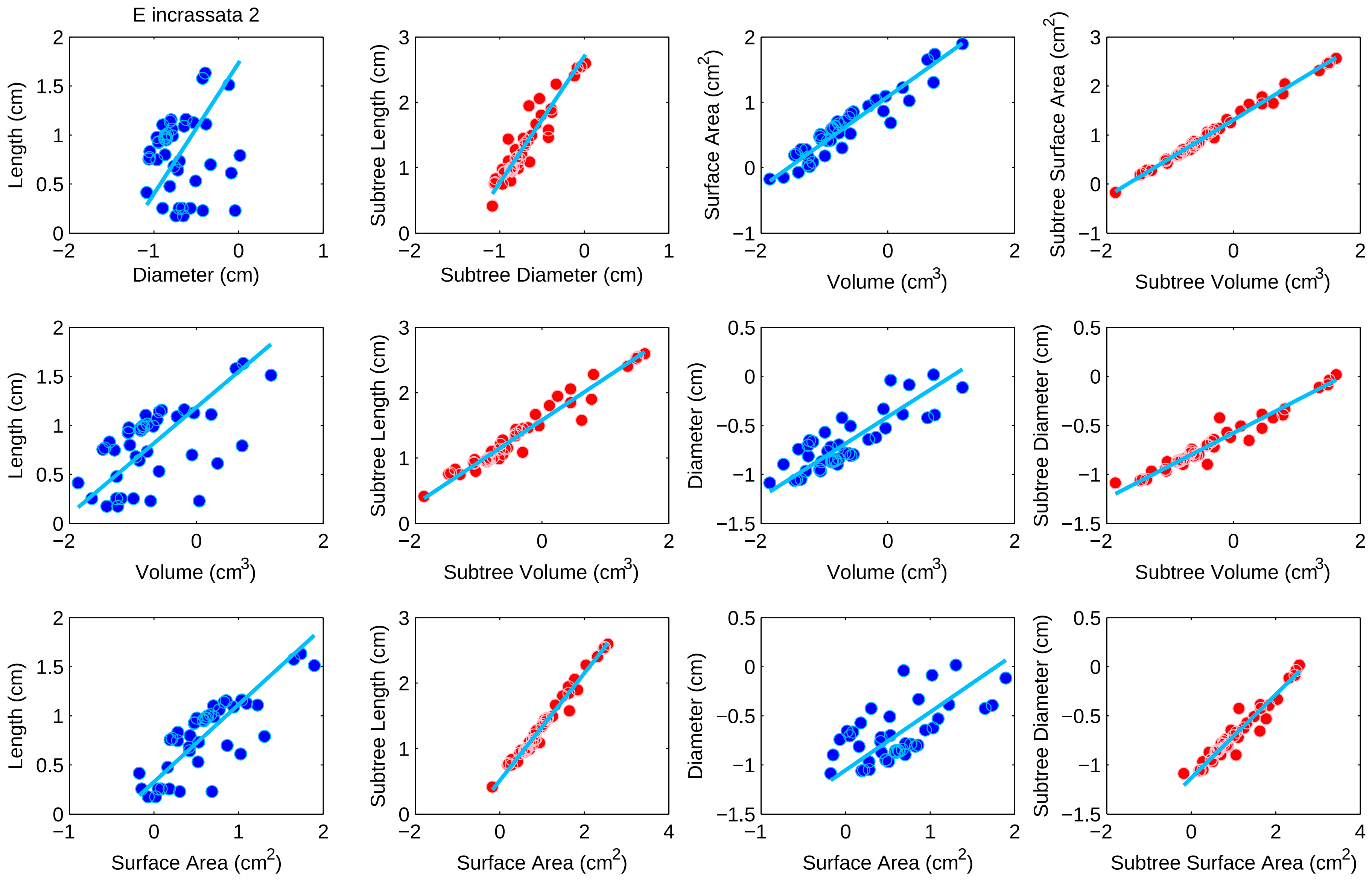

Figure S17. Allometric relationships for E. incrassata sample 2. Sapling dimensions for raw data (blue symbols) and subtrees (red symbols).

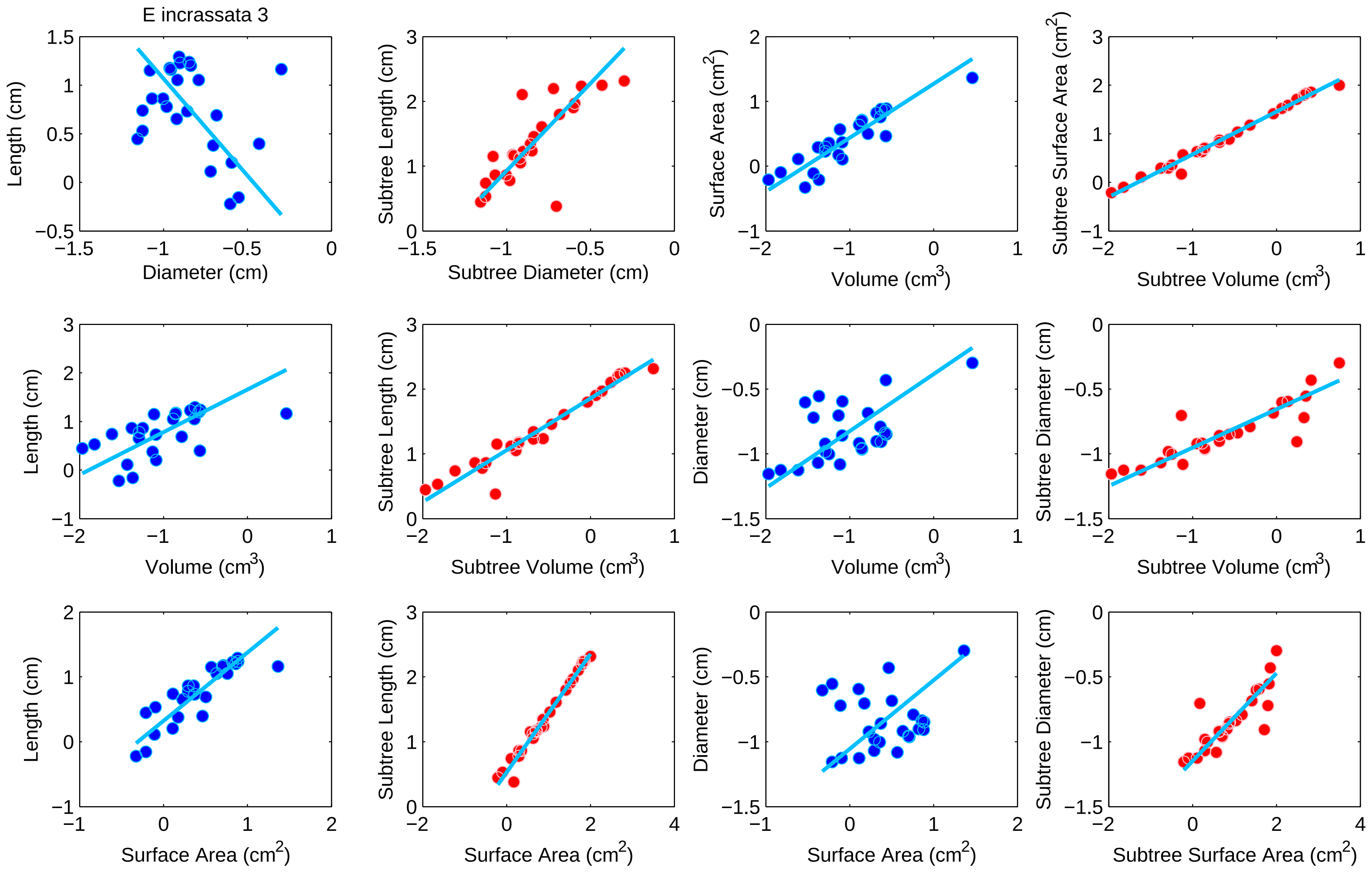

Figure S18. Allometric relationships for E. incrassata sample 3. Sapling dimensions for raw data (blue symbols) and subtrees (red symbols).

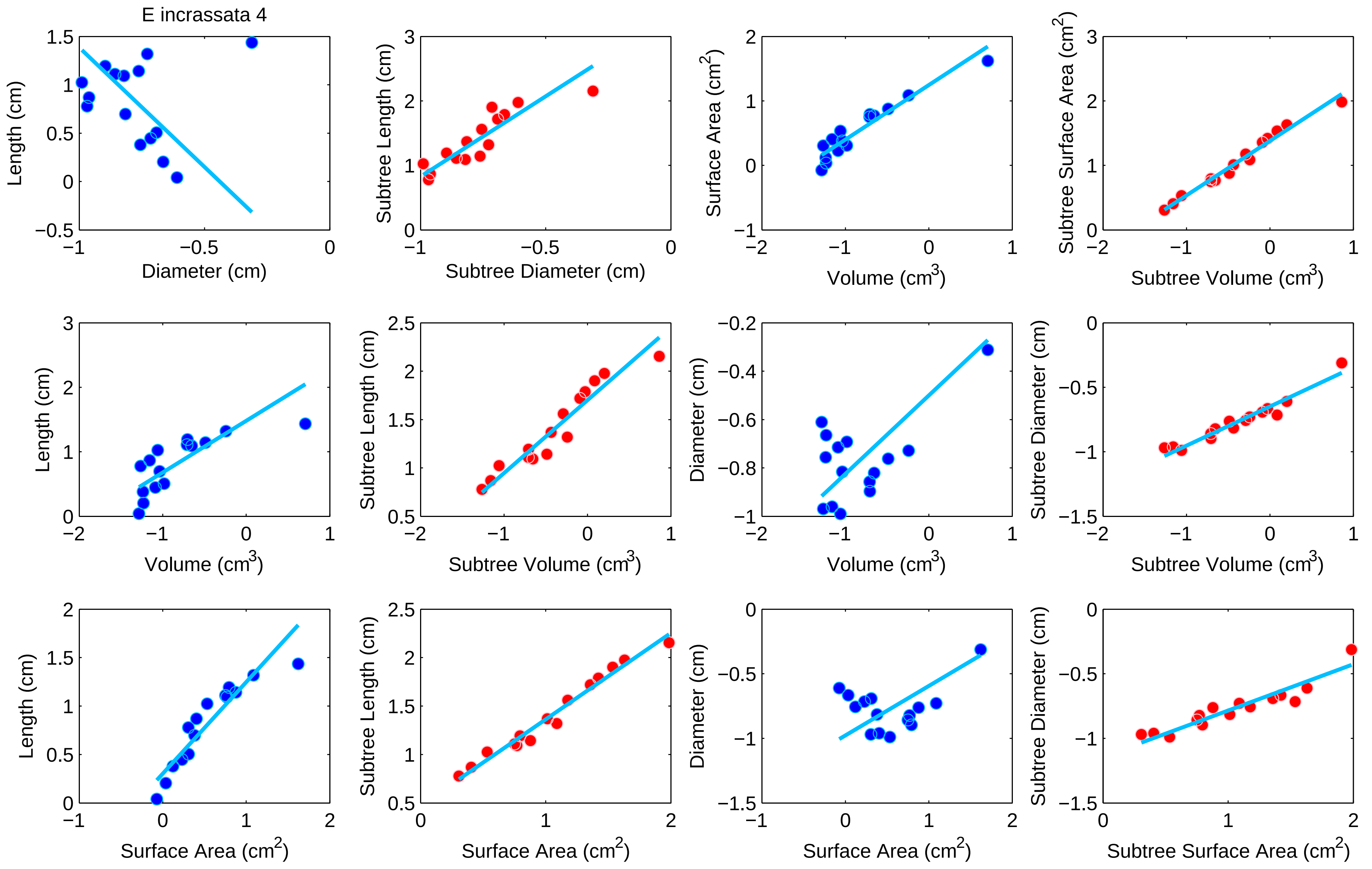

Figure S19. Allometric relationships for E. incrassata sample 4. Sapling dimensions for raw data (blue symbols) and subtrees (red symbols).
